# Identification of a chaperone-code responsible for Rad51-mediated genome repair

**DOI:** 10.1016/j.jbc.2024.107342

**Published:** 2024-05-03

**Authors:** Khushboo Rani, Akanksha Gotmare, Andreas Maier, Ruchira Menghal, Nashat Akhtar, Nupur Fangaria, Johannes Buchner, Sunanda Bhattacharyya

**Affiliations:** 1 Department of Biotechnology and Bioinformatics, School of Life Sciences, University of Hyderabad, Hyderabad, Telangana, India; 2 Department of Chemistry, Technical University of Munich, Garching, Germany

**Keywords:** chaperone, heat shock protein 90 (Hsp90), DNA repair, *Saccharomyces cerevisiae*, proteostasis, homologous recombination

## Abstract

Posttranslational modifications of Hsp90 are known to regulate its *in vivo* chaperone functions. Here, we demonstrate that the lysine acetylation-deacetylation dynamics of Hsp82 is a major determinant in DNA repair mediated by Rad51. We uncover that the deacetylated lysine 27 in Hsp82 dictates the formation of the Hsp82-Aha1-Rad51 complex, which is crucial for client maturation. Intriguingly, Aha1-Rad51 complex formation is not dependent on Hsp82 or its acetylation status; implying that Aha1-Rad51 association precedes the interaction with Hsp82. The DNA damage sensitivity of Hsp82 (K27Q/K27R) mutants are epistatic to the loss of the (de)acetylase *hda1Δ*; reinforcing the importance of the reversible acetylation of Hsp82 at the K27 position. These findings underscore the significance of the cross talk between a specific Hsp82 chaperone modification code and the cognate cochaperones in a client-specific manner. Given the pivotal role that Rad51 plays during DNA repair in eukaryotes and particularly in cancer cells, targeting the Hda1-Hsp90 axis could be explored as a new therapeutic approach against cancer.

Cells are continuously exposed to various endogenous and exogenous factors that lead to different forms of DNA lesions. One of the most detrimental damages is DNA double-strand break (DSB), which, if left unrepaired, leads to cell death. The homologous recombination (HR)-mediated DNA repair is one of the two major DNA repair mechanisms that maintains genome integrity (1). In lower eukaryotes, such as budding yeast, the high-fidelity HR-mediated DNA repair mechanism is favored over the error prone nonhomologous end joining pathway. The HR-mediated DNA break repair is accomplished by various repair proteins, and one of the key recombinase proteins is Rad51 that governs the homology search, strand invasion, and DNA strand exchange. In *Saccharomyces cerevisiae*, after the initial processing of the DSBs, the formation of Rad51 nucleoprotein filaments at the broken ends are mediated by Rad52, Rad54, and Rad55/Rad57 heterodimer (2, 3, 4).

Hsp90 is a molecular chaperone that is evolutionarily conserved and is known to stabilize the client proteins associated with different cellular processes. Hsp90 has been linked with the DNA damage response and repair pathways (5, 6). Studies have elucidated that the inhibition of Hsp90 disrupts the HR-mediated repair of DSBs formed due to ionizing radiation exposure (7, 8). Moreover, it has been observed that Hsp90 inhibition hampers the activation of Ataxia-Telangiectasia mutated (ATM) and the function of MRE11-RAD50-NBS1 (MRN) complex in response to ionizing radiation (9). A recent study in *S. cerevisiae* has illustrated that Hsp82, an ortholog of human Hsp90 alpha isoform, is accumulated in the nucleus and is recruited at the damaged site upon elicitation of a single DSB (10). Another study has revealed that Hsp82 modulates the stability of Chl1, a sister chromatid cohesion protein, such that the inhibition of Hsp82 by 17-AAG leads to decreased association of sister chromatids and enhanced prevalence of chromosomal loss (11). Furthermore, it was reported that the strain harboring an ATPase dead mutant of Hsp82, experiences extreme sensitization to DNA mutagenic agents along with the destabilization of Rad51 and Rad52 (12). It was observed that although deleting the charged linker region of Hsp82 does not hamper Rad51 stability, but it reduces methyl methane sulfonate (MMS)-dependent Rad51 nuclear foci formation, and impairs Rad51 activity during HR (12, 13). Although it is known that Hsp82 regulates the stability and activity of Rad51, but the comprehensive understanding of the mechanism behind this regulation remains obscure.

The Hsp90 chaperone machinery is also regulated by various posttranslational modifications (PTMs); some of which have been identified to play a regulatory role in DNA repair pathway. A study revealed that Hsp90 is phosphorylated at Thr7 by DNA-pyruvate kinase_cs_ upon DNA damage and is recruited to the DSBs (14). Another study has depicted that ionization induced ATM-mediated phosphorylation of the nuclear pool of Hsp90 at its Thr5/Thr7 residues, but ATM is crucial for maintaining γH2AX levels in the damaged chromatin (15). In yeast, modification of Hsp90 phosphorylation sites in the middle domain and the C-terminal domains increased the sensitivity toward UV radiation (16). Hsp90 PTMs are known to modulate Hsp90 chaperone function by altering its ATPase activity, binding with cochaperone and client proteins, subcellular localization, and inhibitor susceptibility (17). Thus, this extensive and combinative array of Hsp90 PTMs subsequently affect several cellular processes. Nevertheless, the biological function and significance of many reported PTM sites of Hsp90 still remain elusive and need to be validated for their effect on Hsp90 chaperone activity.

Reversible acetylation has been implicated as a crucial regulatory PTM of Hsp90 that influences the function of Hsp90 chaperone. The set of enzymes accountable for these modifications are: histone acetyltransferases, that insert an acetyl group to the lysine residues; and histone deacetylases (HDACs), that remove the acetyl group from the modified lysine residues. Studies have demonstrated that the inhibition of HDACs induces hyperacetylation of Hsp90 leading to its reduced binding with ATP and client proteins (18, 19). The inhibition of HDAC6, a class II histone deacetylase, obstructs the association of Hsp90 with glucocorticoid receptor and aryl hydrocarbon receptor, and consequently disrupts the complex formation and activity of these receptor proteins (19, 20, 21). In mammalian systems, it has been reported that Hsp90 is acetylated at K294 residue which directs the Hsp90 chaperone cycle by regulating the interaction of cochaperones and client proteins with Hsp90 (22). Another study in yeast has demonstrated that Hsp82 is acetylated at K27 position, and the inhibition of lysine deacetylases results in compromised stability and function of calcineurin: a fungal drug resistance modulator (23). Albeit, the reversible acetylation of Hsp90 has been recognized to impact diverse cellular activities, but whether this PTM of Hsp90 has any role in DNA repair pathway has not been addressed so far.

In this study, we have identified a distinct chaperone code of Hsp82 which is crucial for the regulation of HR-mediated DNA repair in yeast. We have compared that acetylation-deacetylation dynamics of lysine residues present in the N-terminal and middle domain of Hsp82 and deciphered the mechanism behind the maturation and activity of Rad51. Here, we report that deacetylation of N-terminal domain 27-lysine of Hsp82 allows the association of Aha1-Rad51 complex to Hsp82. Absence of the HDACs or the presence of acetylation mimetic Hsp82 mutant prohibits effective Rad51 maturation and sensitizes the cells to DNA damaging agents.

## Results

### K27 acetylation/deacetylation mimicking *hsp82* mutants are susceptible toward DNA damaging agents

**Figure 1:**
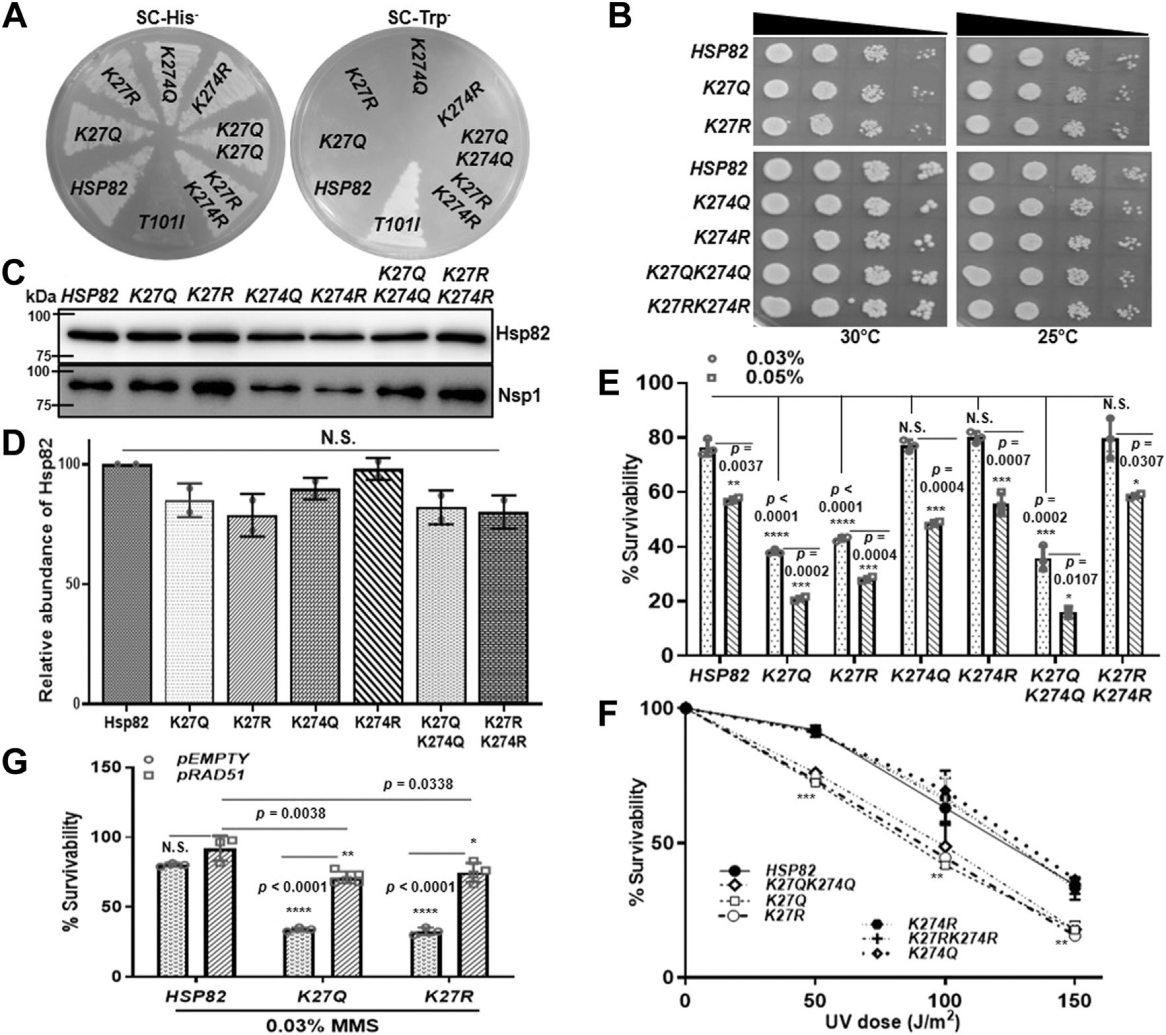
**K27 acetylation/deacetylation mimicking *hsp82* mutants are susceptible to DNA damaging agents.***A*, generation of K27 and K274 acetylation/deacetylation mimicking *hsp82* mutant strains by plasmid shuffling; *T101Ihsp82* strain was used as a negative control for conforming the strain generation. *B*, serially diluted spotting of WT and *hsp82* mutant strains shows no growth defect in the mutant *hsp82* strains; cells were grown at two different temperatures *i.e.*, 30 °C (*left panel*) and 25 °C (*right panel*). *C*, Western blot showing the stability of Hsp82 in the acetylation/deacetylation mimicking mutant *hsp82* strains; Nsp1 was used as a loading control. *D*, quantification of two independent repeats of the above experiment shows no change in the steady state level of mutant Hsp82 proteins; the mean value (±SD) was plotted; *p* values were calculated using the two-tailed Student’s *t* test (NS, not significant). *E*, return to growth assay depicting the cell survivability of acetylation/deacetylation mimicking *hsp82* mutant strains upon MMS treatment. Cells were treated with 0.03% and 0.05% of MMS, and percentage survivability was calculated relative to that of the untreated cells. The above experiment was conducted three independent times, and the mean value (±SD) was plotted; *p* values were calculated using the two-tailed Student’s *t* test (∗∗∗∗*p* < 0.0001, ∗∗∗*p* < 0.001 ∗∗*p* < 0.01, ∗*p* < 0.05, NS, not significant). *F*, percentage survivability of the *hsp82* mutant strains after treatment with three different doses of UV radiations *i.e.*, 50 J/m^2^, 100 J/m^2^, and 150 J/m^2^. The mean value (±SD) of three independent repeats of the above experiment was plotted; *p* values were calculated using the two-tailed Student’s *t* test (∗∗∗*p* < 0.001, ∗∗*p* < 0.01). *G*, Rad51 overexpression causes partial reversal of MMS sensitivity in *K27Qhsp82* and *K27Rhsp82* strains; however, it showed no survival advantage once overexpressed in WT strain. The above experiment was conducted three independent times, and the mean value (±SD) was plotted; *p* values were calculated using the two-tailed Student’s *t* test (∗∗∗∗*p* < 0.0001, ∗∗*p* < 0.01, ∗*p* < 0.05, NS, not significant). MMS, methyl methane sulfonate.

Earlier it was shown that human Hsp90 alpha isoform is acetylated at the 294th lysine (K294) residue, which is conserved as 274th lysine (K274) residue of yeast (*S. cerevisiae*) Hsp82 (22). Mass spectrometry analysis identified that 27th lysine (K27) residue of Hsp82 remains acetylated in *hda1Δrpd3Δ* strain (23). We compared various orthologs of Hsp90 from different organisms and found that both the K27 and K274 residues of Hsp82 are evolutionary conserved within eukaryotes (Fig. S1, *A* and *B*). According to the domain structure of Hsp82, the K27 residue is present in the N-terminal domain that contains the ATPase domain of Hsp82, while the K274 residue lies at the junction of the charged linker region and the middle domain (Fig. S1*C*). To understand the effect of acetylated Hsp82 in DNA repair pathway, we generated six point-mutations (Fig. 1*A*) to mimic the acetylated (K to Q) or unacetylated (K to R) lysine, as used in previous studies (22, 23). The respective mutants as well as WT *HSP82* were cloned individually in a low-copy expression vector (His^+^). The vectors harboring the mutants were transformed in a yeast strain (24) where *HSP82* and *HSC82* were deleted at the chromosomal loci, harboring an episomal copy of *HSP82* on a low-copy plasmid bearing Trp^+^ selectable marker. The isogenic mutant and WT strains were generated by growing them for several generations in the presence of tryptophan to ensure plasmid (His^+^) shuffling. Finally, their growth in histidine dropout media and lack of growth in tryptophan dropout media confirmed the absence of WT Hsp82 (Trp^+^) plasmid and the presence of mutant *hsp82* in each of the strains (Fig. 1*A*). As a control, yeast cells (24) harboring *T101Ihsp82* on a Trp^+^ plasmid was streaked on histidine or tryptophan dropout plates. We examined the growth pattern of the aforementioned strains at 30 °C and 25 °C by spotting the serially diluted cells on yeast extract, peptone, dextrose (YPD) plates. We observed that the mutant *hsp82* strains showed comparable growth at both the temperatures with respect to the isogenic WT strain (Fig. 1*B*). We wanted to investigate the steady state level of all the aforementioned hsp82 mutant proteins. The Western blot showed similar level of Hsp82 in all the mutant strains (Fig. 1*C*). The nucleoskeletal-like protein Nsp1 was shown as a loading control, as used in our earlier studies (10). Quantification of Western blot images from independent set of experiments depicted a comparable expression of hsp82 mutant proteins as compared to the WT (Fig. 1*D*). Earlier studies from our laboratory showed that loss of Hsp82 function sensitizes the cells toward DNA damaging agents (12). To investigate whether the acetylation-deacetylation status of K27 and K274 residues of Hsp82 has any impact on DNA repair pathway, we exposed the mutant strains to 0.03% and 0.05% of MMS (a DNA alkylating agent) and observed that all the strains displayed a dose-dependent sensitivity (Fig. 1*E*). We observed that the *K27Qhsp82*, *K27Rhsp82*, and *K27QK274Qhsp82* exhibited more susceptibility toward MMS compared to the isogenic WT strain; however, *K274Qhsp82, K274Rhsp82*, and *K27RK274Rhsp82* displayed comparable sensitivity to that of the WT (Fig. 1*E*). Next, we exposed the strains to three different doses of UV radiations namely; 50 J/m^2^, 100 J/m^2^, and 150 J/m^2^. The UV radiation induces the formation of thymidine dimers in the DNA. These may lead to the formation of DNA DSBs when the replication fork passes through that (25). We found that *K27Qhsp82*, *K27Rhsp82*, and *K27QK274Qhsp82* mutant strains exhibited significant reduction in cell survivability in a dose-dependent manner. On the other hand, the *K274Qhsp82*, *K274Rhsp82*, and *K27RK274Rhsp82* mutant strains showed comparable sensitivity as that of the WT strain (Fig. 1*F*). Together, we conclude that mutations (Q/R) in K274th position of Hsp82 do not affect MMS and UV sensitivity unlike the K27th position. In the double mutant *K27RK274Rhsp82*, the effect of *K27R* mutation was compensated by introduction of mutation at *K274R* and as a result, the strain behaved as that of the isogenic WT control. Nevertheless, *K27Qhsp82* mutant phenotype prevailed in the double mutant strain *K27QK274Qhsp82*. Previously we have shown that Hsp82-dependant Rad51 stability is essential for survivability of the cells in presence of MMS (12). We wanted to test whether *Rad51* overexpression suppresses the MMS sensitivity in K-27 *hsp82* mutants. We transformed Rad51 overexpression plasmid in *K27Qhsp82* and *K27Rhsp82* strains and exposed them to 0.03% MMS. We observed that in both the mutant strains, *Rad51* overexpression partially reverses the MMS sensitivity (Fig. 1*G*, left panel). As a control we generated *Rad51*-overexpression strain by transforming *Rad51* in WT strain and performed MMS sensitivity assay. The strain behaved similarly to the WT strain, not giving any survival advantage upon *Rad51* overexpression. Thus, we conclude that acetylation status of K27 modulates the Hsp82 dependent genome stability in yeast through Rad51.

### Aha1 can stimulate the ATPase activity of K27 acetylation/deacetylation mutants of hsp82

**Figure 2:**
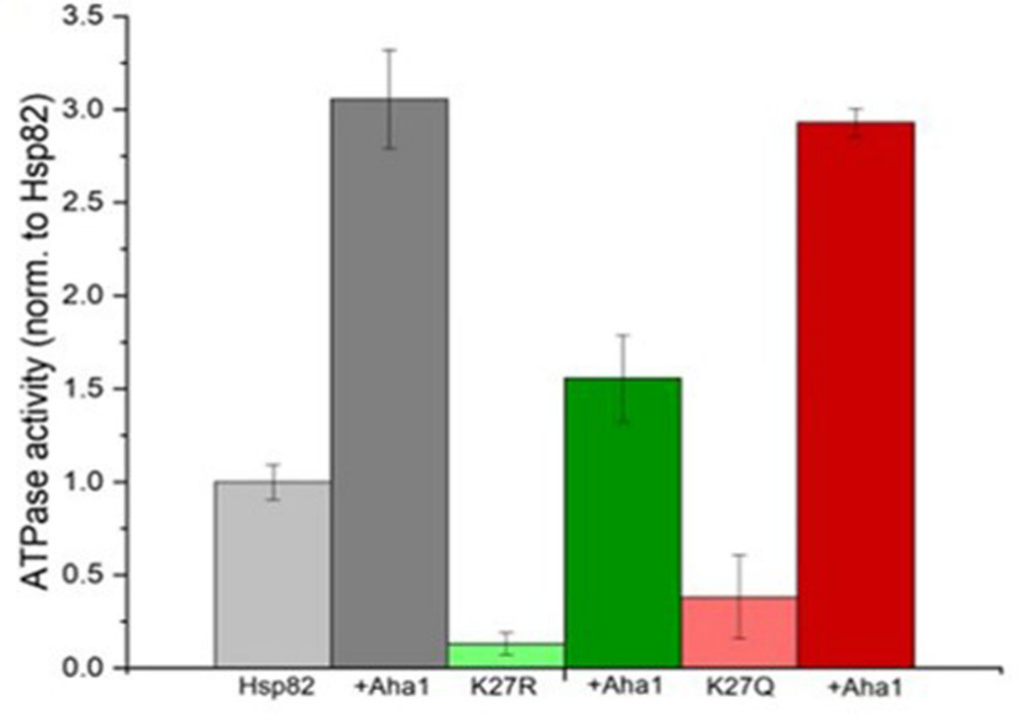
**Aha1 can stimulate the ATPase activity of K27 acetylation/deacetylation mutants of hsp82.** Hsp82, Hsp82 K27R, and Hsp82 K27Q ATPase activities were determined in the presence or absence of Aha1. Hsp82 ATPase and addition of Aha1 are shown in *light* and *dark gray*, Hsp82 K27R and addition of Aha1 in *light* and *dark green* and Hsp82 K27Q and addition of Aha1 in *light* and *dark red*. The ATPase activities were normalized.

We intended to measure the effect of K27Q and K27R mutation on the Hsp82 ATPase. To that end, we purified both the mutant proteins and compared their ATPase activity with that of the WT protein. We observed that both the mutants showed defects in ATPase activity (Fig. 2). Between the two mutants, Hsp82^K27R^ showed about two-fold reduction in ATPase activity compared to the Hsp82^K27Q^ mutant. We wanted to determine whether Aha1 can activate the ATPase activity of the mutants as that of WT Hsp82. In the case of the Hsp82^K27Q^ mutant, although the inherent ATPase activity of the mutant was two-fold less compared to the WT, the Aha1-induced ATPase activity was similar to that of the WT (Fig. 2). In the case of Hsp82^K27R^ mutant, we observed that there was an enhanced ATPase activity in the presence of Aha1, albeit it was considerably less compared to the WT (Fig. 2). Thus, *in vitro* assays clearly showed that both the mutants were able to form a functional complex with Aha1, as seen by the stimulation of their inherent ATPase activity.

### Hda1 deletion is epistatic to the K27 acetylation/deacetylation mimicking Hsp82 mutants

**Figure 3:**
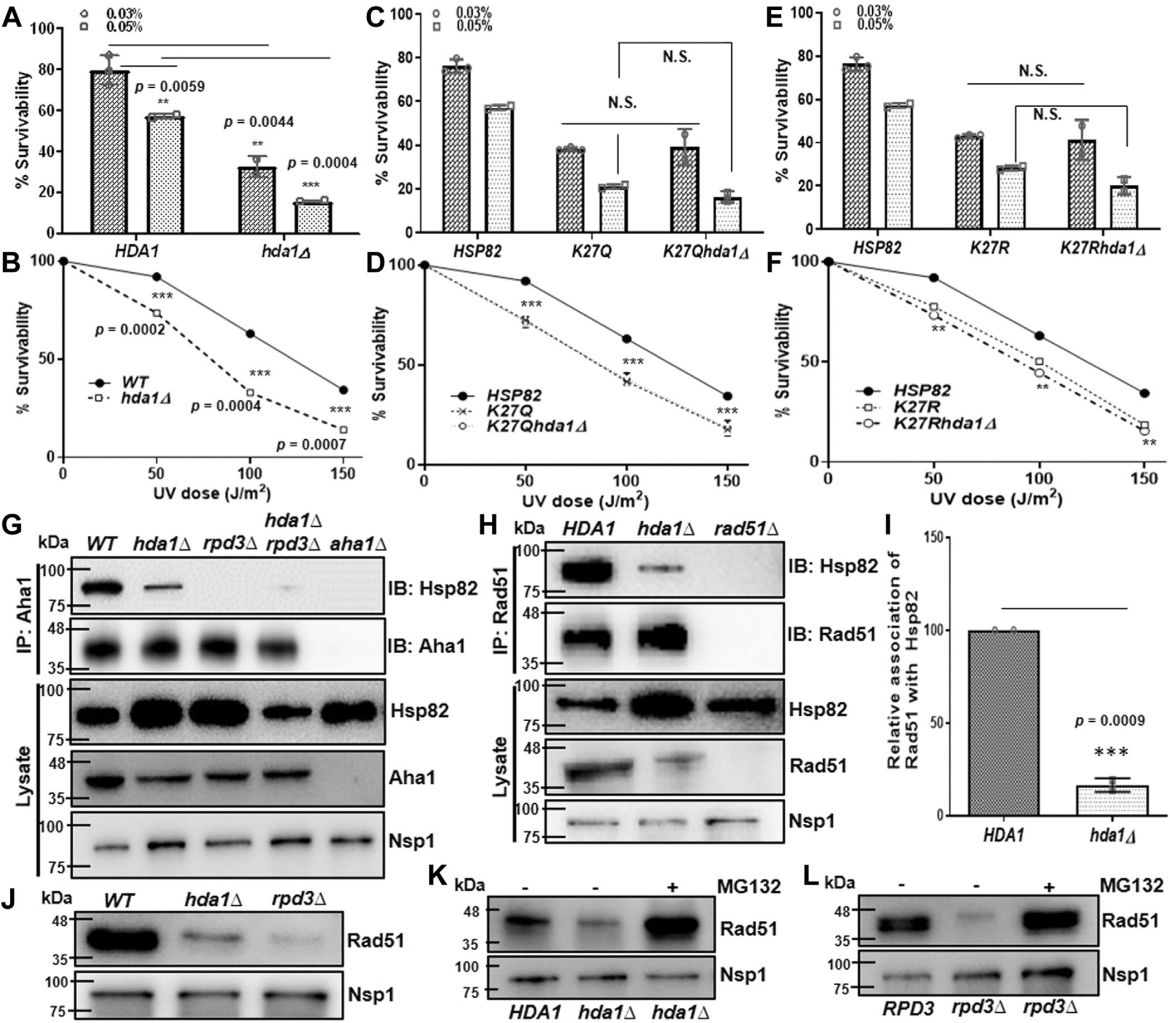
**Hda1 deletion is epistatic to the K27 acetylation/deacetylation mimicking Hsp82 mutants.***A*, return to growth assay depicting the cell survivability of *hda1Δ* strain upon 0.03% MMS and 0.05% MMS. The percentage survivability was calculated relative to that of the untreated cells. The above experiment was conducted three independent times, and the mean value (±SD) was plotted; *p* values were calculated using the two-tailed Student’s *t* test (∗∗∗*p* < 0.001, ∗∗*p* < 0.01). *B*, percentage survivability of *hda1Δ* strain after treatment with 50 J/m^2^, 100 J/m^2^ and 150 J/m^2^ of UV doses. The mean value (±SD) of three independent repeats of the above experiments was plotted; *p* values were calculated using the two-tailed Student’s *t* test (∗∗∗*p* < 0.001). *C*, percentage survivability of *K27Qhsp82hda1Δ* strain with respect to *K27Qhsp82* strain upon 0.03% MMS and 0.05% MMS treatment. The above experiment was conducted three independent times, and the mean value (±SD) was plotted; *p* values were calculated using the two-tailed Student’s *t* test (NS, not significant). *D*, percentage survivability of *K27Qhsp82hda1Δ* strain after treatment with 50 J/m^2^, 100 J/m^2^, and 150 J/m^2^ of UV doses. The mean value (±SD) of three independent repeats were plotted; *p* values were calculated using the two-tailed Student’s *t* test (∗∗∗*p* < 0.001). *E* and *F*, percentage survivability of *K27Rhsp82hda1Δ* strain showed similar dose-dependent sensitivity to *K27Rhsp82* strain upon exposure to various doses of MMS and UV radiations. The above experiments were conducted three independent times, and the mean value (±SD) was plotted; *p* values were calculated using the two-tailed Student’s *t* test (∗∗*p* < 0.01, NS, not significant). *G*, Aha1 protein was immunoprecipitated with Aha1 specific antibody, from WT, *hda1Δ*, *rpd3Δ*, and *hda1Δrpd3Δ* strains, the pellet was probed for the presence of Hsp82 in each fraction. The expression of Aha1 and Hsp82 were probed in each strain and presented in the *bottom* panel as lysate. *H*, Western blots showing CoIP of Rad51 with Hsp82 in WT and *hda1Δ* strains. *I*, the above experiment was repeated two times and the quantification of Western blots showed a moderate reduction in interaction of Rad51 with Hsp82 in the absence of Hda1; the mean value (±SD) was plotted; *p* values were calculated using the two-tailed Student’s *t* test (∗∗∗*p* < 0.001). *J*, Western blot showing the stability of Rad51 in *hda1Δ* and *rpd3Δ* strains. *K*, Western blot showing the endogenous level of Rad51 in *hda1Δ* strain in presence and absence of proteasome-inhibitor MG132. *L*, Western blot showing the endogenous level of Rad51 in *rpd3Δ* strain in presence and absence of proteasome-inhibitor MG132. CoIP, coimmunoprecipitation; MMS, methyl methane sulfonate.

The biochemical study of the mutants does not explain the DNA damage sensitivity of the K27 mutants as observed in Figure 1, *E* and *F*. In order to test our hypothesis that the moderate DNA damage sensitivity of *K27Q/K27R* mutants of Hsp82 was indeed due to alteration of the acetylation-deacetylation dynamics of the chaperone, we investigated the effect of HDACs on the Hsp82-chaperoned DNA repair pathway. Both the Class I (Rpd3) and Class II (Hda1) HDACs were reported to deacetylate K27 of Hsp82 (23). Mammalian homolog of Hda1 (HDAC6) was shown to regulate the Hsp90 chaperone function by deacetylating K294-Hsp90 (22). However, there is no report till date which shows whether human Rpd3 ortholog HDAC3, also regulates the chaperone function of Hsp90. Hence, we primarily wanted to focus whether Hda1-induced Hsp82 acetylation influences the DNA repair pathway. To study this, we checked the DNA damage sensitivity of *hda1* deleted strain in the presence of two different DNA damaging agents. We treated the *hda1Δ* strain with 0.03% and 0.05% of MMS and observed that Hda1 deletion sensitized the strains moderately toward MMS in a dose-dependent manner (Fig. 3*A*). The *hda1Δ* strain also displayed a dose-dependent decrease in survivability upon UV treatment (Fig. 3*B*). Next, we examined whether the deletion of *hda1* is epistatic to the acetylation/deacetylation mimicking the mutation of *hsp82* at K27. To this end, we knocked out *hda1* from *K27Qhsp82* and *K27Rhsp82* strains. We found that upon treatment with 0.03% and 0.05% of MMS, the cell survivability of *K27Qhsp82hda1Δ* strain was reduced in a dose-dependent manner and followed similar trend as that of the *K27Qhsp82* strain (Fig. 3*C*). Likewise, the *K27Qhsp82hda1Δ* strain displayed similar kind of UV sensitivity as that of the *K27Qhsp82* strain (Fig. 3*D*). The *K27Rhsp82hda1Δ* strain also exhibited similar kind of susceptibility toward MMS and UV treatments as that of the *K27Rhsp82* strain (Fig. 3, *E* and *F*). To decipher the mechanism of this phenomenon, we wanted to determine whether the intracellular chaperone function of Hsp82 is supported by Hda1. Since, our earlier study revealed that ATPase activating cochaperone Aha1 regulates the HR-mediated nuclear function of Hsp82 (10), we measured the extent of *in vivo* Hsp82 association with Aha1 in the absence of each of the two different lysine deacetylases namely Hda1 and Rpd3. We performed Aha1 pull-down in the WT, *hda1Δ*, *rpd3Δ*, and *hda1Δrpd3Δ* strains and observed that the intracellular complex formation between Aha1 and Hsp82 was decreased drastically in all three mutant strains (Fig. 3*G*). Further, between the two single histone deacetylase deletion strains, *rpd3Δ* strain manifested complete loss of Hsp82-Aha1 association. Rad51, a major recombinase of yeast has been established earlier as a direct client of Hsp82 (12). The study revealed that Rad51 remained associated with Hsp82, and inhibition of Hsp82 led to the proteasomal degradation of Rad51 (12). We measured whether the loss of association between Hsp82 and Aha1 in Hda1 deleted strain, has any impact on the intracellular complex formation between Rad51 and Hsp82. We observed that the level of Rad51 protein is considerably low in *hda1Δ* strain. In order to capture Hsp82-Rad51 interaction, we have overexpressed Rad51 from an episomal plasmid and immunoprecipitated Rad51. We observed that the intracellular association of Rad51 with Hsp82 was diminished significantly in the null *hda1* strain (Fig. 3*H*), compared to the WT strain. We repeated the experiment and found that the association of Rad51 with Hsp82 was decreased by 5-fold in *hda1Δ* strain (Fig. 3*I*). Consequently, the amount of Rad51 in the lysate (Fig. 3*H*, second lane from bottom) was significantly reduced in *hda1Δ* strain, although it harbored Rad51 overexpression plasmid. We observed that Rad51 stability was reduced in both *hda1Δ* as well as in *rpd3Δ* strain (Fig. 3*J*), where Rad51 expression was maintained by its own promoter. Interestingly, the stability of Rad51 was more affected in *rpd3Δ* strain than in *hda1Δ* strain. To verify whether the loss of Rad51 in the absence of HDACs was due to the loss of Hsp82 chaperone function, resulting in its proteasomal degradation, we measured the amount of Rad51 in the presence of a proteasome inhibitor, MG132. We observed the restoration of Rad51 levels upon MG132 treatment in both *hda1Δ* (Fig. 3*K*) and *rpd3Δ* strains (Fig. 3*L*). Together, we conclude that the K27 acetylation mimicking mutant of *hsp82* is epistatic to *hda1Δ*, and that the deacetylation of Hsp82 at lysine 27th position is mandatory for its association with Aha1. Subsequently, the loss of lysine deacetylases (Hda1/Rpd3) inhibits Hsp90 chaperone activity and causes proteasomal degradation of Rad51.

### Intracellular complex formation between Rad51 and Hsp82 is facilitated by Aha1

**Figure 4:**
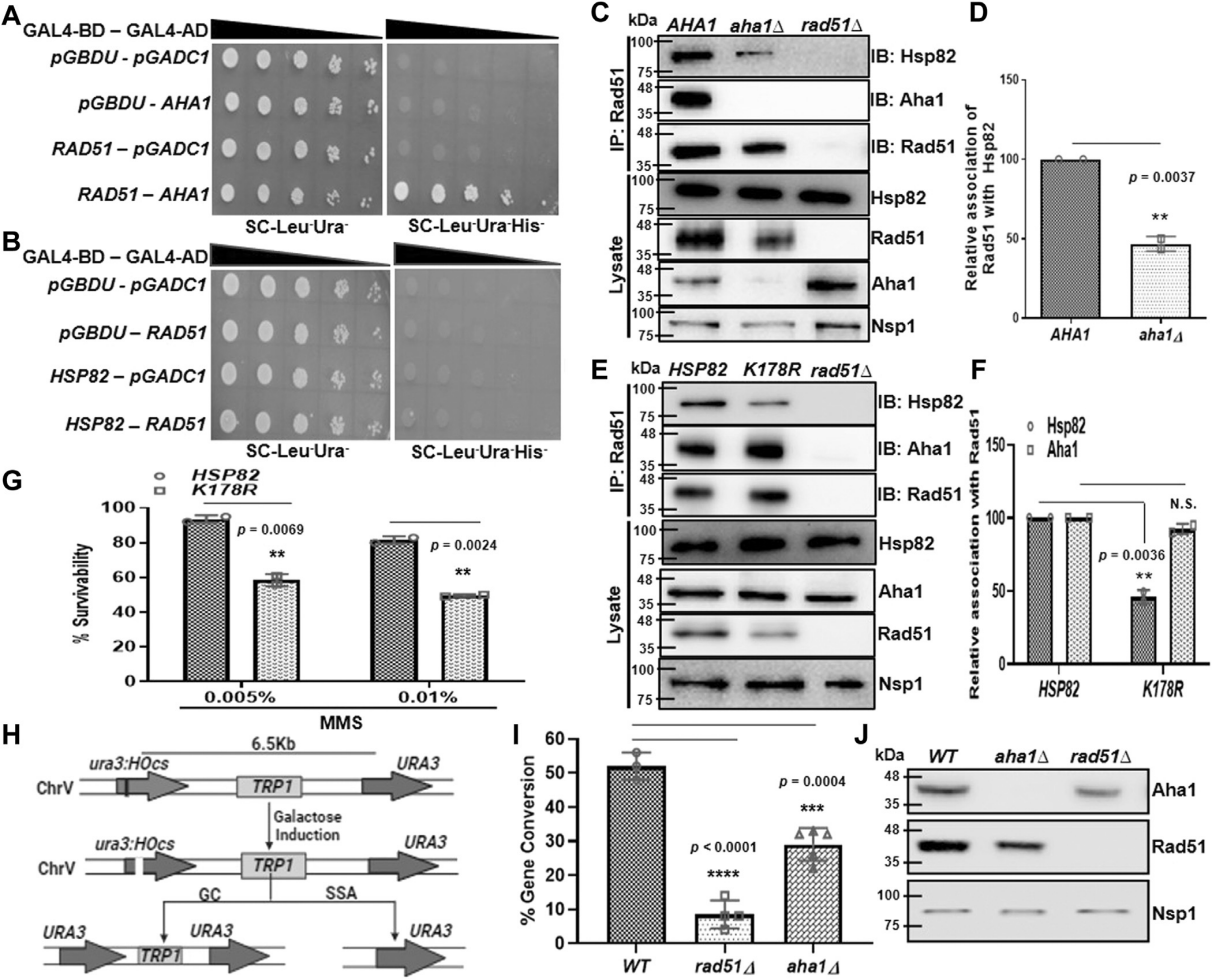
**Intracellular complex formation between Rad51 and Hsp82 is facilitated by Aha1.***A*, yeast two-hybrid assay depicting the interaction of Rad51 with Aha1. Strains harboring varied bait and prey vectors were grown till 0.5 absorbance and serially diluted. The diluted cells were spotted on SC-Leu⁻Ura⁻ (*left* panel), and SC-Leu⁻Ura⁻His⁻ to assess the interaction (*right* panel). *B*, yeast two-hybrid analysis showed no physical interaction of Rad51 with Hsp82. *C*, Western blots showing CoIP of Rad51 with Hsp82 and Aha1 in WT and *aha1Δ* strains. *D*, the above experiment was repeated two times and quantification of Western blots depicted a two-fold reduction in the interaction of Rad51 with Hsp82 in the absence of Aha1; the mean value (±SD) was plotted; *p* values were calculated using the two-tailed Student’s *t* test (∗∗*p* < 0.01). *E*, Western blots showing CoIP of Rad51 with Hsp82 and Aha1 in WT and *K178Rhsp82* strains. *F*, the quantification of Western blot images showed significant reduced association between Rad51 and Hsp82 in *K178Rhsp82* strain, while Rad51 interaction with Aha1 remained unchanged; the mean value (±SD) was plotted; *p* values were calculated using the two-tailed Student’s *t* test (∗∗*p* < 0.01). *G*, return to growth assay depicting the cell survivability of WT and *K178Rhsp82* strain upon 0.005%- and 0.01%-MMS treatment. The above experiment was conducted in two independent sets, and the mean value (±SD) was plotted; *p* values were calculated using the two-tailed Student’s *t* test (∗∗*p* < 0.01). *H*, schematic presentation behind the principle of gene conversion assay in *NA3* strain. The cell harbors two copies of *URA3* in chromosome V, one of which has a *HO* endonuclease cut site incorporated within it. Upon induction of *HO*, the single DSB will be created and it can be repaired either by the Rad51 dependent gene conversion (GC), utilizing *URA3* template that is 6 kb apart or by the Rad51 independent single strand annealing (SSA). In GC-mediated DNA repair, the intermediate *TRP* cassette is retained, however; the *TRP* cassette is deleted in SSA mediated DNA repair. *I*, percent gene conversion efficiency was plotted for the individual strain. The experiment was repeated more than three times and the mean value (±SD) was plotted; *p* values were calculated using the two-tailed Student’s *t* test (∗∗∗∗*p* < 0.0001, ∗∗∗*p* < 0.001). *J*, Western blot confirms the deletion of Aha1 in *NA3 aha1Δ* strain. Rad51 levels were compared between the *NA3* and *NA3 aha1Δ* strain. CoIP, coimmunoprecipitationl; DSB, double-strand break; MMS, methyl methane sulfonate.

The above result prompted us to decipher whether the intracellular complex formation between Rad51 and Hsp82 is dependent on the *in vivo* Aha1-Hsp82 complex formation. We hypothesized that Rad51 protein might need the presence of Aha1 for its optimum association with Hsp82. To test our hypothesis, we determined whether there is a direct interaction of Rad51 with Aha1 as well as Rad51 with Hsp82. We performed yeast-two hybrid assay to examine the physical association of Aha1/Hsp82 with Rad51. We observed a weak physical interaction between Aha1 and Rad51, as together they could activate the *HIS3* reporter gene (Fig. 4*A*). However, Rad51 did not show any interaction with Hsp82 (Fig. 4*B*). We performed the coimmunoprecipitation (CoIP) assay in WT and *aha1Δ* strain and compared the presence of Hsp82 in the Rad51 pull-down fractions. To our surprise, we found that in null *aha1* strain, Hsp82 level was significantly reduced (Fig. 4*C*) in the Rad51 immunoprecipitation (IP) fraction. We repeated this assay and the Image J (https://imagej.net/ij/download.html) analysis of independent sets of experiments showed that the relative intra-cellular association between Rad51 and Hsp82 was reduced by 2-fold in the absence of Aha1 (Fig. 4*D*). Consequently, we observed that Rad51 stability was also severely reduced in *aha1Δ* strain, as seen in the lysate (third panel from the bottom, Fig. 4*C*). To establish further that Aha1 promotes the complex formation between Hsp82 and Rad51, we used a well-characterized non-SUMOylate N-terminal mutant (K178R) of Hsp82 that had been reported to prevent intracellular interaction with Aha1 (26). We performed CoIP in the strain harboring *K178Rhsp82* as the sole copy of Hsp82, and investigated the extent of *in vivo* complex formation between Rad51 and Hsp82^K178R^. We used Rad51 antibody to pull-down Rad51 from both the WT and the mutant strains and compared the level of Hsp82 associated with the pellet. We observed that the level of Hsp82^K178R^ in the Rad51 pull-down fraction was reduced drastically compared to that of the WT (Fig. 4*E*, first lane from top); although, Aha1 association to Rad51 remained unaltered (Fig. 4*E*, second lane from top). We estimated the relative intracellular association between Rad51 and Hsp82/Aha1 in *K178Rhsp82* strain by repeating the CoIP assay and plotted (Fig. 4*F*). We found that there was a 2-fold reduction in Rad51-Hsp82^K178R^ association in *K178Rhsp82* mutant strain compared to the WT, although the same between Rad51 and Aha1 remained unperturbed (Fig. 4*F*). The lysate showed comparable expression of Hsp82^K178R^ protein as that of the WT; although, the stability of Rad51 was significantly reduced in *K178Rhsp82* strain (Fig. 4*E*, second panel from bottom). Thus, our study revealed that *in vivo* association of Aha1-Hsp82 is the prerequisite for effective intracellular complex formation between Hsp82 and its client Rad51, which in turn is necessary for stability of Rad51 protein. We validated this result by monitoring the DNA damage sensitivity of *K178Rhsp82* strain. We subjected *K178Rhsp82* strain to two different doses of MMS (0.005% and 0.01%) and measured its survivability. We observed that the mutant strain displayed increased sensitivity toward MMS compared to the WT (Fig. 4*G*). Overall, we observed that there was nearly 50% reduction in cell survivability in *K178Rhsp82* mutant strain compared to WT in each dose of MMS. We intended to see whether Aha1 deletion causes any reduction in Rad51 function. To that end, we compared Rad51-dependant gene conversion efficiency between WT and *aha1Δ* strain. The principle underlying the assay is represented schematically in Figure 4*H*. The WT *NA3* (27) harbors two consecutive *URA3* genes, one of which is mutated by incorporation of *HO* endonuclease site within it. The induction of *HO* endonuclease by galactose creates a DSB at the *ura3* mutant locus, which can be repaired by the donor *URA3* that is situated 6 kb away from the *HO* cut site. We determined the % GC in *NA3*, *NA3 rad51Δ* (27) and *NA3 aha1Δ* and observed a significant difference in their gene conversion efficiencies. While the *NA3* and *NA3 rad51Δ* showed 52% and 8.5% GC efficiencies respectively, *NA3 aha1Δ* showed 29% GC efficiency, which is almost 50% less compared to the WT (Fig. 4*I*). The Western blot confirms the absence of Aha1 in *NA3 aha1Δ* strain (Fig. 4*J*). We observed a significant reduction in the endogenous level of Rad51 in *NA3aha1Δ* strain (Fig. 4*J*).

### Reversible acetylation of K27 regulates Hsp82 interaction with cochaperones

**Figure 5:**
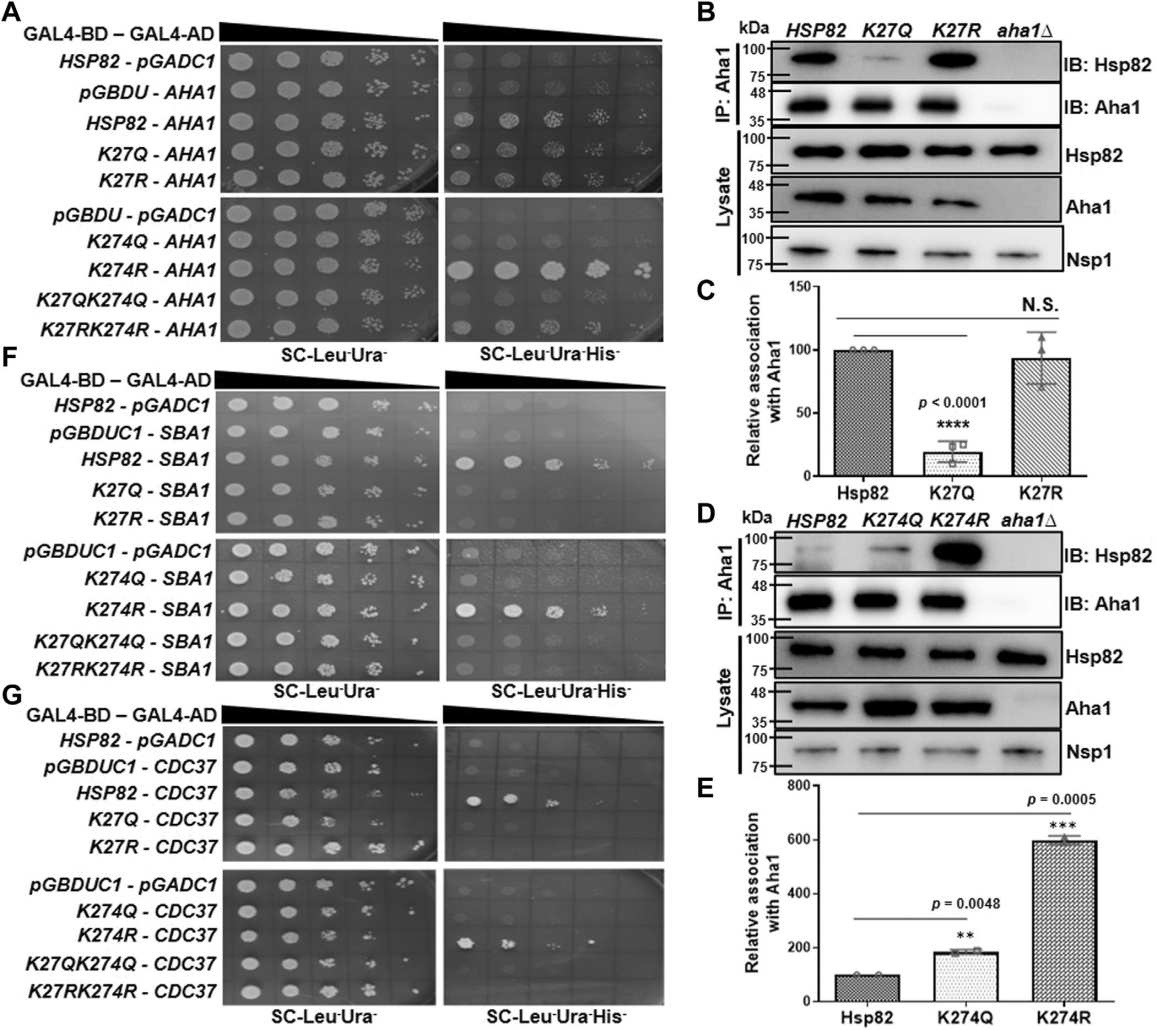
**Reversible acetylation of K27 regulates Hsp82 interaction with cochaperones.***A*, yeast two-hybrid assays were performed to evaluate the interaction between Aha1 and each of the six acetylation/deacetylation mimicking Hsp82 mutants by plating the serially diluted cells in histidine dropout media. *B*, Aha1 was immunoprecipitated from WT as well as from *K27Qhsp82* and *K27Rhsp82* strains and the binding of Hsp82 and its mutants were detected by immunoblotting. *C*, the above experiment was repeated and the quantification of Western blots showed 5-fold reduced complex formation between Aha1 and Hsp82^K27Q^, although the same with Hsp82^K27R^ remained unchanged; the mean value (±SD) was plotted; *p* values were calculated using the two-tailed Student’s *t* test (∗∗∗∗*p* < 0.0001). *D*, Aha1 was immunoprecipitated from WT as well as from *K274Qhsp82* and *K274Rhsp82* strains. *E*, the quantification of two independent repeats of the above experiment depicted enhanced association of Aha1 with both *hsp82K274Q* and *hsp82K274R* mutants; the mean value (±SD) was plotted; *p* values were calculated using the two-tailed Student’s *t* test (∗∗*p* < 0.01, ∗∗∗*p* < 0.001). *F* and *G*, yeast two-hybrid assays were performed to evaluate the interaction between Sba1 and Cdc37, respectively to each of the six acetylation/deacetylation mimicking Hsp82 mutants.

Previously it was shown that, Aha1 association with the middle domain of Hsp82 triggers a conformational change to the N-terminal nucleotide binding domains of Hsp82, such that, the two protomers are oriented in close proximity to form a dimer; which in turn enhances the binding between C-terminal domain of Aha1 with the N-terminal Hsp82 dimer (28). We hypothesize that the PTM of Hsp82 K-27 might impact the intracellular complex formation between Aha1 and Hsp82. To that end, we used both *in vitro* (yeast two-hybrid assay) and *in vivo* assays (CoIP) to estimate their interaction. We observed that Hsp82^K27Q^, Hsp82^K27R^, and Hsp82^K27RK274R^ displayed similar kind of interaction with Aha1, as that of WT Hsp82; however, Hsp82^K274R^ displayed slightly better growth in histidine dropout plate (Fig. 5*A*). We further measured the ability of Aha1 to form intracellular complex with the above-mentioned mutants of Hsp82 through CoIP assay. We observed that while the intracellular complex formation between Hsp82^K27Q^ and Aha1 was significantly attenuated, the association between Hsp82^K27R^ and Aha1 remained similar to that of the WT (Fig. 5*B*). The experiment was repeated several times and we quantified about 5-fold reduced association between Aha1 and Hsp82^K27Q^ (Fig. 5*C*). In case of K274 mutation, a moderately increased association between Aha1 and Hsp82^K274R^ was observed. Aha1 and Hsp82^K274Q^ also displayed a slight increase in association (Fig. 5, *D* and *E*). We also probed the interaction between the aforementioned mutants of Hsp82 with two other cochaperones Sba1 and Cdc37, which were previously coupled to the DNA repair pathway (29, 30). We observed that out of the six mutants, Hsp82^K274R^ showed comparable growth in the histidine dropout media confirming a comparable interaction with Sba1 (Fig. 5*F*) and Cdc37 (Fig. 5*G*) relative to the WT protein. However, all the other mutants were defective in mediating interaction with Sba1 or Cdc37 (Fig. 5, *F* and *G*). Together, our study has suggested that the acetylation mimicking K27 mutation of Hsp82 is severely defective in intracellular complex formation with Aha1 and in mediating direct physical association with two major cochaperones Sba1 and Cdc37. We also conclude that K274R mutant shows significantly stronger association with all the three above-mentioned cochaperones relative to the WT protein.

### Acetylation mimicking K-27 mutant negatively impacts Hsp82 interaction with Rad51

**Figure 6:**
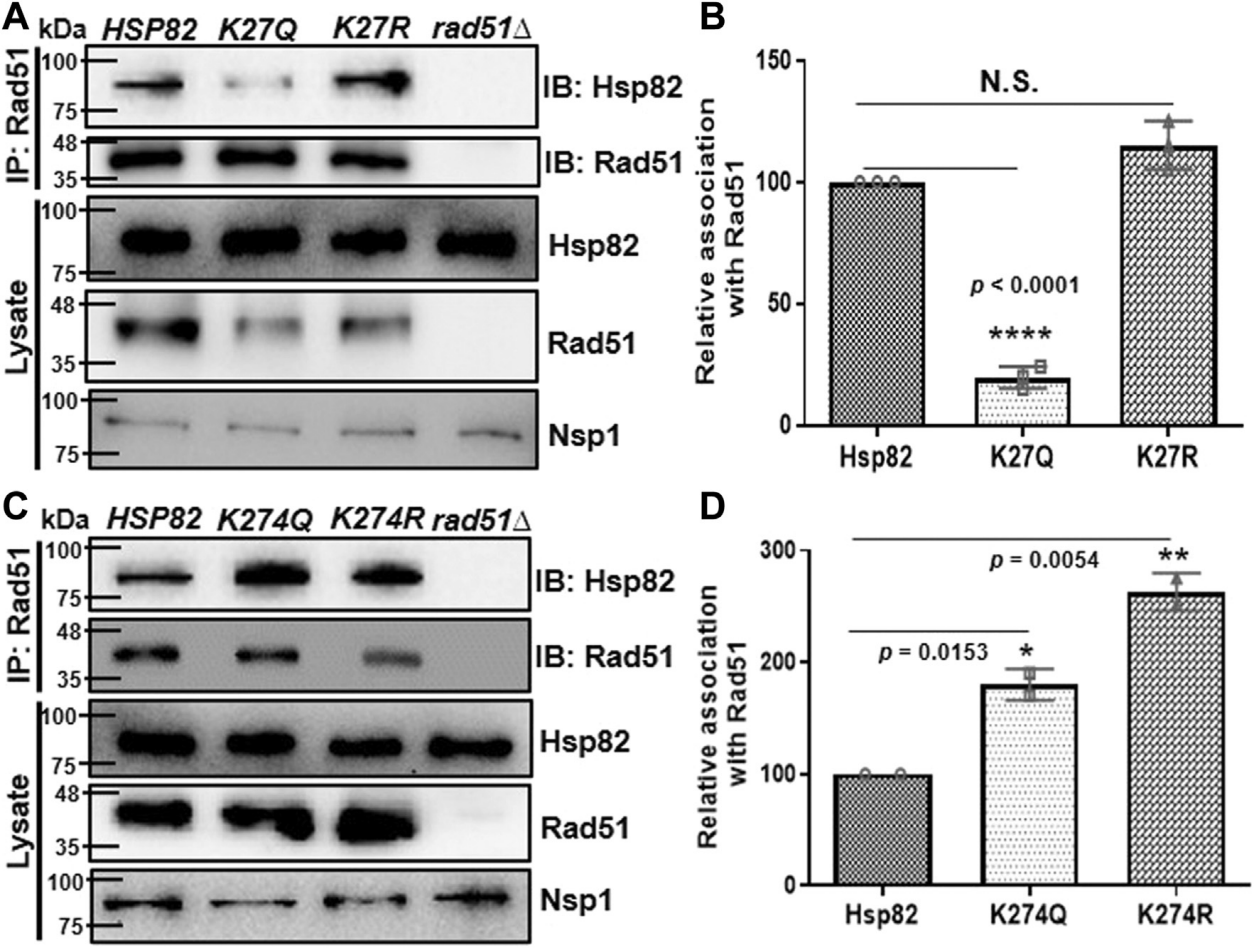
**Acetylation mimicking K-27 mutant impacts Hsp82 interaction with Rad51.***A*, Rad51 was immunoprecipitated from WT, *K27Qhsp82*, and *K27Rhsp82* strains. The extent to which Hsp82 and its mutants were associated with Rad51 was detected by immunoblotting; *Δrad51* was used as a negative control for Rad51 IP. *B*, the above experiment was repeated, and the quantification of Western blots showed a four-fold reduction in association of Rad51 with *hsp82K27Q* mutant, whereas the interaction was unaltered with Hsp82^K27R^ mutant; the mean value (±SD) was plotted; *p* values were calculated using the two-tailed Student’s *t* test (∗∗∗∗*p* < 0.0001, NS, not significant). *C*, Rad51 was immunoprecipitated from WT, *K274Qhsp82*, and *K274Rhsp82* strains; the extent of association of Hsp82 and its mutants with Rad51 were detected by immunoblotting. *D*, the quantification of independent repeats of the above experiment showed enhanced association of Rad51 with both *hsp82K274Q* and *hsp82K274R* mutants; the mean value (±SD) was plotted; *p* values were calculated using the two-tailed Student’s *t* test (∗∗*p* < 0.01, ∗*p* < 0.05).

As we observed that the intracellular complex formation of Aha1 with Hsp82^K27Q^ was moderately reduced, we hypothesized that the extent of Rad51 association with the aforementioned Hsp82 mutants would be variable accordingly. We over-expressed Rad51 in different *hsp82* single mutant strains and immunoprecipitated Rad51 with anti-Rad51 antibody. We observed that Hsp82^K27R^ and WT Hsp82 protein were immunoprecipitated with Rad51 to a similar extent. However, Rad51 displayed a drastic reduction in interaction with Hsp82^K27Q^ mutant (Fig. 6*A*). We repeated the experiment and quantified that Hsp82^K27Q^ mutant displayed a 4-fold reduced association with Rad51, while there was no significant difference in the association between Hsp82^K27R^ and Rad51 (Fig. 6*B*). Importantly, we observed that Rad51 displayed reduced stability in *K27Qhsp82* strain, as seen in the whole cell lysate (second panel from the bottom, Fig. 6*A*) even under Rad51 overexpression condition. On the other hand, Rad51 association with Hsp82^K274Q^ was slightly enhanced and that with Hsp82^K274R^ was moderately enhanced (Fig. 6*C*), which are consistent according to their respective association with Aha1 (Fig. 6, *D* and *E*). The Image J analysis of Western blot prepared from independent sets of experiments showed that there was a 1.7-fold and 2.5-fold stronger interaction between Rad51 and Hsp82^K274Q^, Hsp82^K274R^, respectively compared to the WT protein (Fig. 6*D*). Together, we conclude that the acetylation status of Hsp82 K27 regulates its association with Rad51.

### K-27 is critical for Hsp82-dependent stability and activity of Rad51

**Figure 7:**
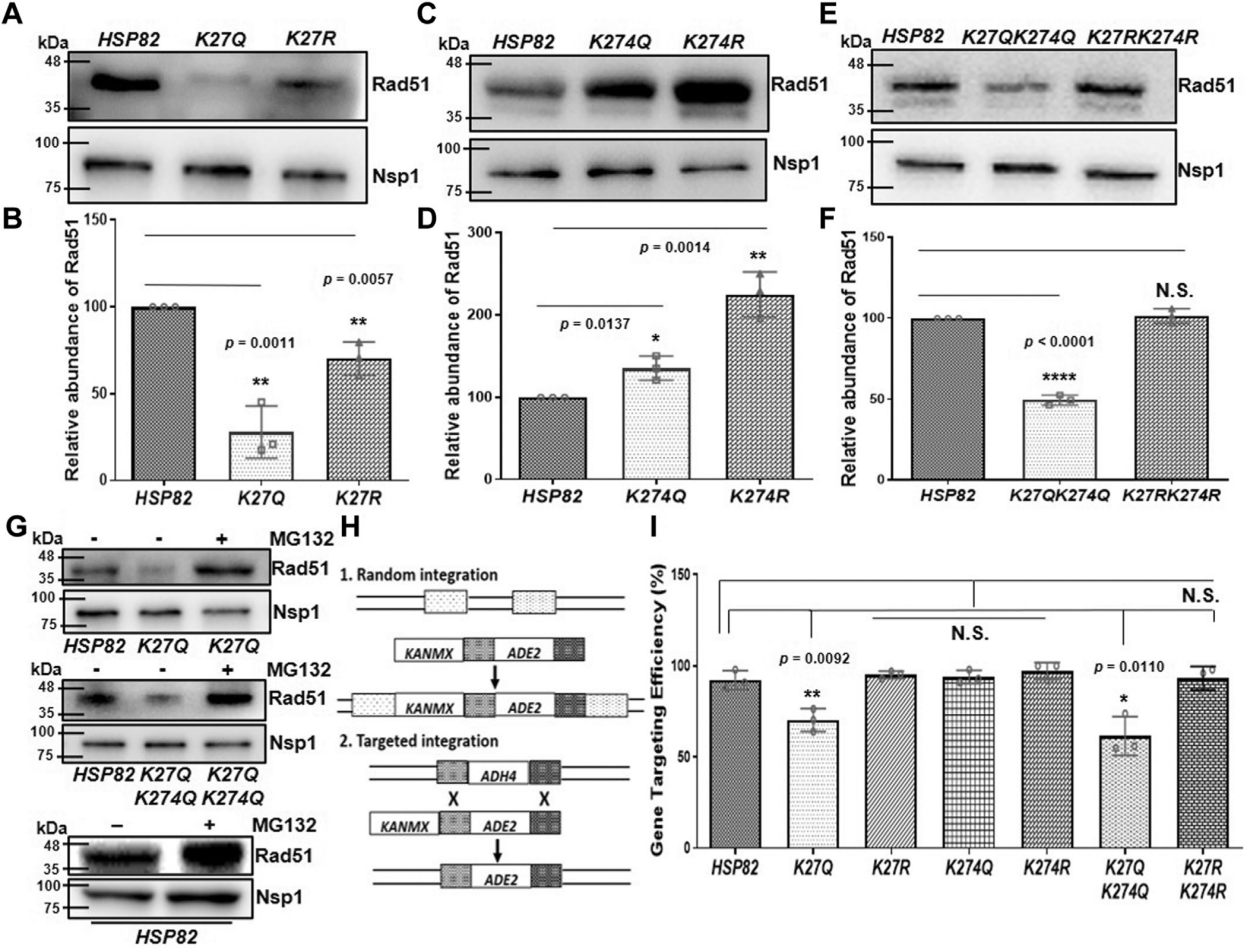
**K-27 is critical for Hsp82-dependent stability and activity of Rad51.***A*, Western blot showing Rad51 level in whole cell extracts of WT, *K27Qhsp82*, and *K27Rhsp82* strains. *B*, the above experiment was repeated and the quantification of Western blots showed a four-fold reduced stability of Rad51 in *K27Qhsp82* strains; the mean value (±SD) was plotted; *p* values were calculated using the two-tailed Student’s *t* test (∗∗*p* < 0.01). *C*, Western blot showing Rad51 level in whole cell extracts of WT, *K274Qhsp82*, and *K274Rhsp82* strains. *D*, the quantification of independent repeats of the above experiment showed increased stability of Rad51 in both *K274Qhsp82* and *K274Rhsp82* strain; the mean value (±SD) was plotted; *p* values were calculated using the two-tailed Student’s *t* test (∗∗*p* < 0.01, ∗*p* < 0.05). *E*, Western blot showing Rad51 level in whole cell extracts of WT, *K27QK274Qhsp82*, and *K27RK274Rhsp82* strains. *F*, the above experiment was repeated and the quantification of Western blots depicted a two-fold reduced stability of Rad51 in *K27QK274Qhsp82* strain, whereas that remained unchanged in *K27RK274Rhsp82* strain; the mean value (±SD) was plotted; *p* values were calculated using the two-tailed Student’s *t* test (∗∗∗∗*p* < 0.0001, NS, not significant). *G*, Rad51 levels in *K27Qhsp82* (*upper* panel), *K27QK274Qhsp82* (*middle* panel), and WT strains (*bottom* panel) were monitored in presence of MG132. *H*, schematic representation of the gene targeting cassette consisting the upstream and downstream homologous stretches of *ADH4* gene, flanking the *ADE2* gene, and a *KANMX* selectable marker. Random integration generates Ade^+^G418S^R^ colonies, while targeted integration results in Ade^+^G418S^S^ colonies. *I*, gene targeting assay showed 30% and 40% reduced gene targeting efficiency in *K27Qhsp82* and *K27QK274Qhsp82* strains, respectively, whereas there was no change in gene targeting efficiency in other *hsp82* mutant strains; the mean value (±SD) was plotted; *p* values were calculated using the two-tailed Student’s *t* test (∗∗*p* < 0.01, ∗*p* < 0.05, NS, not significant).

Previously, we have established Rad51 as a client of Hsp82, and we observed that the lack of Hsp82 function leads to proteasomal degradation of Rad51 with complete loss of Rad51 activity (12). We sought to investigate whether the acetylation status of K27/K274 had any impact on Rad51 stability and its activity. We isolated the total protein from three independent batches of the aforementioned single and double mutant strains and probed for the endogenous level of Rad51. One representative image for each set of mutants was presented (Fig. 7, *A, C* and *E*). We estimated the relative abundance of Rad51 from each of the mutant strains by Image J analysis and observed that the stability of Rad51 was decreased severely (by 4-fold) in *K27Qhsp82* and showed a little reduction (1.3-fold) in *K27Rhsp82* mutant strains (Fig. 7*B*). In case of K274 acetylation/deacetylation mimicking *hsp82* mutant strains, we found that although the stability of Rad51 was slightly increased in *K274Qhsp82*, its stability was moderately enhanced (2-fold) in *K274Rhsp82* mutant strain (Fig. 7*D*). In case of the double mutant strains, Rad51 stability was decreased in *K27QK274Qhsp82* strain by 2-fold; however, the stability of Rad51 remained unchanged in *K27RK274Rhsp82* strain (Fig. 7*F*). We reason that as *K274Q* mutation promotes slightly higher stability of Rad51. Hence, the reduced level of Rad51, as observed in the double K-Q mutant, is due to the mutation in *K27Q* position of Hsp82. However, in case of double K-R mutant, since *K274R* mutation provides two-fold higher stability of Rad51 compared to the WT, the little defect in Rad51 stability, as seen in the *K27R* mutant strain, is compensated in the double K-R mutation. As a result, the strain harbors comparable level of Rad51 as that of the WT. To prove that Rad51 was misfolded and hence had undergone proteasomal degradation in *K27Qhsp82* (Fig. 7*G* upper panel) and in *K27QK274Qhsp82* (Fig. 7*G* middle panel) strains, we measured Rad51 levels after treating the strains with MG132 and observed the restoration of Rad51 in those strains. In a parallel experiment, we investigated the level of Rad51 in MG132-treated WT strain and observed that MG132 prevents the proteasomal degradation of Rad51 in these cells (Fig. 7*G*, bottom panel). To assess the activity of Rad51 in the aforementioned *hsp82* mutants, we performed gene targeting assay that depends on the HR mechanism of the cell, governed by active Rad51. For this, we used a targeting cassette that consists of the upstream and downstream homologous stretches of *ADH4* gene, flanking the *ADE2* gene, and a *KANMX* selectable marker such that, if there is a random integration, then the whole cassette will be incorporated at any arbitrary locus in the genome (31) (upper panel, Fig. 6*H*) resulting in Ade^+^G418^R^ colonies. Conversely, when there is a HR–mediated targeted integration, then the cassette will get incorporated at the *ADH4* locus and the *KANMX* selectable marker (lower panel, Fig. 7*H*) will be lost, resulting in Ade^+^G418^S^ colonies. Our study revealed that both the *K27Qhsp82* and *K27QK274Qhsp82* mutants exhibited about (30–40) % reduced gene targeting efficiency, respectively. Intriguingly, the *K27Rhsp82*, *K274Qhsp82*, *K274Rhsp82*, and *K27RK274Rhsp82* mutants showed no significant change in the gene targeting efficiency (Fig. 7*I*). This result concludes that K27 acetylation mimicking mutant shows defective HR recombination due to failure in providing Rad51 stability.

## Discussion

Our present study has unraveled a novel chaperone code of Hsp82 that is critical for Rad51 homeostasis. High-resolution mass spectrometry has identified that human Hsp90 alpha and beta isoform are acetylated on 14th and 4th lysine residues, respectively (32). In a previous study, acetylated-lysine 294 of human Hsp90 alpha isoform was identified to play a regulatory role in the Hsp90 chaperone cycle (22). In yeast, the corresponding lysine residues of Hsc82, that is, K-270 and K-27 were found to be acetylated (23). Mass spectrometry analysis of Hsp82 in *hda1Δrpd3Δ* strain however, could detect only K27-acetylated form (23). We studied the effect of both acetylation mimetic and deacetylation mimetic K27 and K274 (corresponding to K270 of Hsc82) mutants of Hsp82. Our study has deciphered that acetylation-deacetylation dynamics of K274 lysine of Hsp82 does not alter Rad51 stability and activity; however, that of K-27 lysine plays an important regulatory role in Rad51-dependent HR pathway.

In this study, we observed that acetylation of a conserved lysine residue (K-27) of Hsp82, leads to the abrogation of its intracellular association with Aha1. A similar effect was also reported earlier, where the abrogation of Hsp82 (K178) SUMOylation prevented its intracellular association with Aha1, although their *in vitro* binding affinities remained similar to the WT (26). Hsp90-Aha1 interaction has been reported to be a major determinant for *in vivo* client protein stability and its activation. In the case of v-Src kinase, the Hsp90 and Aha1 association was shown to promote the client protein activation (33, 34), whereas, in the case of cystic fibrosis transmembrane conductance regulator protein, the loss of Hsp90-Aha1 association, led to increased stability of cystic fibrosis transmembrane conductance regulator (26). The present study demonstrates that Hsp90-Aha1 association is mandatory for stabilization and activity of Rad51. We established that in Aha1 deletion-strain Rad51 dependent GC efficiency is significantly reduced. Furthermore, in the strain harboring the SUMOylation defective Hsp82^K178R^ mutant, Rad51 fails to be associated with Hsp82 in optimal amount that subsequently affects Rad51 stability and increases the susceptibility of cells toward MMS.

Earlier, we have shown that Aha1 plays a regulatory role in nuclear translocation of Hsp82 upon DNA damage (10). In the absence of Aha1, there is a complete loss of DNA damage-induced nuclear accumulation of Hsp82 (10). Our present study has revealed that Aha1-Hsp82 association regulates the maturation of the major recombinase, Rad51. We speculate that Aha1 facilitates the interaction between partially folded Rad51 and Hsp82 during its chaperone cycle, which eventually results in proper folding of Rad51. When cells are exposed to MMS, the endogenous level of Rad51 increases by several folds (12). For maturation of these increased amount of Rad51, Aha1 is also found to be upregulated (10) upon MMS treatment. Our study indicates that inhibition of Aha1-Hsp90 association can be used as a means to target the DNA repair pathway.

We reason that the DNA damage sensitivity of K-27 mutants of *hsp82* is an outcome of two distinct factors: the altered ATPase activity of mutant hsp82 proteins and an altered association with Aha1 in an acetylation-dependant manner. The Hsp82^K27Q^ protein showed reduced ATPase activity compared to the WT chaperone. However, in the presence of Aha1, it showed an increase in ATPase activity, which is comparable to that of WT protein. Thus, the mutant protein is not defective in binding to Aha1 *in vitro;* the loss of *in vivo* association between Hsp82^K27Q^ and Aha1 is purely dependent on the cellular context, such as acetylation of Hsp82. Nevertheless, the loss of intracellular association between Aha1 and the Hsp82^K27Q^ mutant inhibits Rad51-Hsp82^K27Q^ association, resulting in the proteasomal degradation of Rad51.

In the case of Hsp82^K27R^ mutant, biochemical assay shows that it is severely defective in ATPase activity. In the presence of Aha1, its ATPase activity is enhanced by several folds, but remains significantly lower than the WT Hsp82. As a result, although Hsp82^K27R^ does not show any defect in its intracellular association with Aha1 and with Rad51, due to the defect in its ATPase activity, the endogenous stability of its client Rad51 is decreased (25%) compared to the WT. Such reduction in Rad51 level does not alter Rad51-mediated gene targeting efficiency but has profound effect on repair of genome wide DNA DSB upon MMS and UV treatment.

To further support our inference that acetylation of Hsp82 is responsible for reduced association with Rad51, we used strains lacking lysine deacetylases (*hda1Δ*, *rpd3Δ*, *and hda1Δrpd3Δ*). We observed a significant reduction in intracellular assembly between Hsp82-Aha1 as well as between Hsp82-Rad51 in *hda1Δ* strain, which was further correlated to reduced stability of Rad51. Although both Hda1 and Rpd3 deacetylate Hsp82, but our study indicates that Rpd3 is a better deacetylase than Hda1. We observed that the association between Hsp82 and Aha1 was completely abrogated in *Rpd3* deleted strain, unlike that observed in *Hda1* deleted strain. We reason that in *hda1Δ* strain, Rpd3 alone can erase the acetyl mark of Hsp82 in a better way than that being done by Hda1 in *rpd3Δ* strain. As a result, there is a slightly higher stability of Rad51 in the *hda1Δ* strain compared to *rpd3Δ* strain. We demonstrated that *K27Qhsp82* and *K27Rhsp82* strains were epistatic to *hda1Δ* strain and they manifested similar degrees of susceptibility toward MMS and UV radiations. This type of Hda1-mediated regulation of Hsp82 clients has been observed earlier. It was found that inhibition of lysine deacetylases impaired the interaction between Hsp90 and its client calcineurin, which eventually affected calcineurin activation (23).

An interesting observation of our study is the epistatic interaction between Hda1 and the nonmodifiable mutant of *hsp82*. This can be explained in the following manner. Rad51 stability depends on ATP hydrolysis activity of Hsp82-Aha1 complex. In *K27Rhsp82* mutant, there is an inherent defect in its ATP hydrolysis activity. This mutant does not have any defect on Aha1 association, hence, there is an acceleration of Aha1 dependant ATPase activity in this mutant. However, the resultant ATPase activity of this complex remains much lower than that of WT Hsp82-Aha1 complex, resulting in destabilization of Rad51. On the other hand, Hda1 deletion leads to acetylation of Hsp82 and hence causes disruption of Hsp82-Aha1 complex. This eventually results in decrease in ATPase activity of this complex and hence destabilization of Rad51. Thus, *K27Rhsp82* and *Hda1* deletion are in the same epistatic pathway.

It is interesting to note that Hsp82^K274R^ behaves as a hyperactive mutant, which manifests better physical association with all three cochaperones tested (Aha1, Cdc37, and Sba1) as seen in the yeast two-hybrid analysis. The IP of Aha1 in *K274Rhsp82* strain resulted in moderately increased association of Hsp82^K274R^ with Aha1, which was correlated to similar extent of increased association of Hsp82^K274R^ with Rad51. As Rad51 association with Hsp82 is directly linked with its stability, we found that Rad51 stability was increased largely in that mutant background.

Class I and Class II HDACs (Rpd3 and Hda1) have earlier been shown to influence the DNA repair pathway. A study demonstrated that the inhibition of HDACs led to the acetylation, followed by the degradation of Sae2 exonuclease, which eventually affected the DNA damage response pathway (35). Our study underlines the importance of Hda1 in regulating the Hsp82 chaperone cycle and demonstrates that in the absence of Hda1, the Hsp82 client Rad51 undergoes proteasomal degradation, thereby making the cells susceptible to DNA damaging agents.

Hsp90 is involved in chaperoning multiple clients that are essential in maintaining crucial cellular pathways. Hence inhibiting Hsp90 function causes detrimental effects to overall cell survivability. Our study has demonstrated that Rad51-dependent homologous recombination-mediated DNA repair pathway can be disrupted without inhibiting Hsp90 chaperone function in general. We demonstrate that inhibition of Hsp82-Aha1 association or inhibition of histone deacetylase targets Rad51 toward proteasomal degradation. Our study emphasizes that the target-specific fine-tuning of the Hsp90 chaperone function can be achieved by posttranslational modifiers such as HDACs.

## Experimental procedures

### Plasmids

The sequences of primers used in this study are given in the Table S1. The acetylation/deacetylation mimicking *hsp82* mutants and the non-SUMOylate *hsp82* mutant (26) were generated by site directed mutagenesis. Using yeast genomic DNA as a template, we used splice-overlap-extension PCR to generate the desired mutations and each mutant was subsequently cloned in a centromeric yeast expression vector *pRS313* within the BamHI and SalI restriction sites. For generating *K27Qhsp82, K27Rhsp82, K274Qhsp82, K274Rhsp82*, and *K178Rhsp82* mutations, the primer pairs OSB504/OSB505, OSB506/OSB507, OSB508/OSB509, OSB510/OSB511, and OSB561/OSB562 were used, respectively. The *K27QK274Qhsp82* and *K27RK274Rhsp82* mutants were created by using *K27Qhsp82* and *K27Rhsp82* cloned vectors as template along with OSB508/OSB509 and OSB510/OSB511 primer pairs, respectively. All the cloned products were confirmed by DNA sequencing. All six acetylation/deacetylation mimicking *hsp82* mutants were further subcloned in bait vector *pGBDUC1*, to generate the *pGBDUC1/K27Qhsp82*, *pGBDUC1/K27Rhsp82*, *pGBDUC1/K274Qhsp82*, *pGBDUC1/K274Rhsp82*, *pGBDUC1/K27QK274Qhsp82*, and *pGBDUC1/K27RK274Rhsp82*. The *K27Qhsp82* and *K27Rhsp82* mutants were further subcloned in *pET-28a* vector between BamHI and SalI restriction sites to generate *pET-28a/K27Qhsp82* and *pET-28a/K27Rhsp82* constructs, respectively. The full-length *SBA1* and *CDC37* were amplified by using yeast genomic DNA as template using primer pairs OSB444/OSB445 and OSB612/OSB624, respectively. Each one was subsequently cloned in the prey vector *pGADC1* between BamHI and SalI restriction sites to generate the *pGADC1/SBA1* and *pGADC1/CDC37*. All Y2H clones were confirmed by DNA sequencing. *pGADC1/AHA1* construct was generated by subcloning *AHA1* from *pTA/AHA1* vector (10). The full-length *HSP82* (36) was subcloned in a 2μ yeast expression vector *pLA* to generate the *pLA/HSP82* construct.

### Yeast strains

Strains used in this study have been added in the Table S1. The acetylation/deacetylation mimicking *hsp82* mutant strains and the non-SUMOylate *hsp82* mutant strain were generated by plasmid shuffling. To that end, we transformed *pRS313/HSP82*, *pRS313/K27Qhsp82*, *pRS313/K27Rhsp82*, *pRS313/K274Qhsp82*, *pRS313/K274Rhsp82*, *pRS313/K27QK274Qhsp82*, *pRS313/K27RK274Rhsp82*, *pRS313/K178Rhsp82*, and *pRS313/K178Rhsp82* plasmids bearing a *HIS* selectable marker, individually in *P82a* strain (24) that harbors *HSP82* on a *TRP* plasmid. The transformants were grown on a synthetic media with tryptophan, and lacking histidine. In this manner, we generated *KRAY20*, *KRAY3*, *KRAY4*, *KRAY5*, *KRAY6*, *KRAY7*, *KRAY8*, and *KRAY41* strains. DNA was isolated from each of the newly constructed strain, and the sequence analysis was performed to confirm the presence of *hsp82* mutant genes. For each mutant, we did our analysis with more than three independent clones. For yeast two-hybrid analysis, the *NKY48, NKY50, RMY1, RMY2, RMY3, RMY4, RMY5, RMY6, RMY7, RMY8, RMY9, RMY10, RMY11, RMY12, RMY13, RMY14, RMY15, RMY16, KRAY21, PMY4, KRAY37, PMY7*, and *KRAY40* strains were generated by sequential transformation of the respective bait and prey vectors in the *PJ69-4A* (37) parental strain. To KO *Hda1* from *W303a*, *KRAY20*, *KRAY3*, and *KRAY4* strains, we first amplified a *TRP* cassette from the *pFA6a-TRP* plasmid (38) flanked by a 40 bp homologous stretch of the upstream and downstream regions of the *HDA1*, using the primer pairs OSB376/OSB377. The PCR product was then transformed to the abovementioned strains to generate *hda1Δ* strain. The KO strain was subsequently confirmed by OSB378/OSB377 primer-pair mediated PCR using the genomic DNA of the newly constructed *hda1Δ* strains. In a similar way, to KO Rpd3, a *HIS* cassette (38) flanked by a 40 bp homologous stretch of the upstream and downstream regions of the *RPD3* gene were generated using OSB375/OSB374 primer-pairs. The PCR product was subsequently transformed to *W303a* and *NFY10* strains to create *NFY11* (*rpd3Δ*) and *NFY12* (*hda1Δrpd3Δ*) strains, respectively. The primer set OSB375/OSB374 was used to confirm the *rpd3Δ* strain. To create *KRAY50* and *KRAY52* strains, *pRS313/RAD51* vector was transformed into *W303a* and *NFY10* strains, respectively. To generate *KRAY32*, *KRAY33*, *KRAY34*, *KRAY35*, *KRAY36*, *KRAY44*, and *KRY42*, the *pTA/RAD51* vector was transformed into *KRAY20*, *KRAY3*, *KRAY4*, *KRAY5*, *KRAY6*, *KRAY41*, and *NFY24* (10) strains, respectively. The *pLA/HSP82* vector was transformed to *KRAY50*, *KRAY52*, *W303a*, *NFY10*, *NFY11*, and *NFY12* strains to generate *KRAY51*, *KRAY53*, *KRAY54*, *KRAY55*, *KRAY56*, and *KRAY57* strains, respectively. To KO Aha1 from *NA3* strain, we amplified the KO cassette from *aha1Δ* strain (10) that contains *KANMX* selectable marker flanked by 321 bp upstream homologous box and 344 bp downstream homologous box of Aha1 using the primer pairs OSB275/OSB720. The PCR product was then transformed to the *NA3* strain to generate *AKG1* (*aha1Δ*) strain. The KO strain was subsequently confirmed by PCR as well as by Western blot analysis.

### MMS sensitivity assay

The DNA damage sensitivity of *KRAY20*, *KRAY3*, *KRAY4*, *KRAY5*, *KRAY6*, *KRAY7*, *KRAY8*, *NAY1*, *KRAY48*, *KRAY49*, and *KRAY41* strains toward MMS were tested according to the earlier used protocol (12). In brief, all strains were grown overnight in YPD medium at 30 °C. Next day, secondary cultures were grown till 0.5 *A*_600_ at 30 °C and were subjected to two doses of MMS (0.03% or 0.05%) for 2 hours. Subsequently, the MMS treated cells were washed with deionized water and about 1000 cells were spread on YPD-agar plates. The plates were incubated for 40 h at 30 °C, and the colonies obtained for both treated and untreated conditions were counted. Further, the percentage survivability was calculated using the formula: % Survivability = [(Number of colonies obtained on treated plate) / (Number of colonies obtained on untreated plate)] × 100.

### UV sensitivity assay

*KRAY20*, *KRAY3*, *KRAY4*, *KRAY5*, *KRAY6*, *KRAY7*, *KRAY8*, *NAY1*, *KRAY48*, and *KRAY49* strains were subjected to different doses of UV radiation and their susceptibility were compared as described earlier (12). Briefly, the cells were grown till 0.5 *A*_600_ at 30 °C and approximately 1000 cells were spread on YPD-agar plates. The spread cells were subsequently exposed to 50 J/m^2^, 100 J/m^2^, and 150 J/m^2^ of UV radiation, followed by incubation at 30 °C for 40 h. Later, the percentage survivability was calculated at each dose of UV radiation.

### Gene conversion assay

This assay was carried out in *NA3, NA3 rad51Δ*, and *NA3 aha1Δ* strains. Each strain was grown for several generations on a plate containing glycerol as a sole carbon source. Next, 1000 cells of each strain were spread on galactose containing plate and incubated for 3 to 4 days at 30 °C. Colonies which survived on galactose containing plate were patched on Trp dropout plate and incubated for 24 h. The ratio of the number of colonies grown on the Trp dropout plate to the total number of colonies tested was calculated to determine the percent gene conversion. The assay was performed more than 3 times for each strain and was plotted using GraphPad Prism 8 (https://www.graphpad.com).

### Gene targeting assay

To perform the gene targeting assay, we followed the protocol used earlier in our laboratory (12). Briefly, an integration plasmid (31) was digested with SalI enzyme to generate a fragment that has *ADE2* gene flanked by the *ADH4* upstream and downstream sequences along with a *KANMX6* selectable marker. The cut plasmid (2 μg) was transformed to the following *ade2* auxotroph; *KRAY20*, *KRAY3*, *KRAY4*, *KRAY5*, *KRAY6*, *KRAY7*, *KRAY8*, and *NAY1* followed by selection for *ADE2* expression by growing on SC-Ade⁻ plate. The transformed colonies will result due to either the targeted or random integration of the cassette to the chromosome. The Ade^+^ colonies were further replica plated on G418 sulfate (G418S) containing plates, to score for the one, in which random integration of the cassette had occurred. The number of G418S^R^ colonies was counted and the gene targeting efficiency was calculated using the following formula: % Gene targeting efficiency = [(Number of Ade^+^ colonies – Number of G418S^R^ colonies) / (Number of Ade^+^ colonies)] × 100.

### Yeast two-hybrid assay

The yeast two-hybrid analysis was done as mentioned previously (39). Briefly, *NKY48, NKY50, RMY1, RMY2, RMY3, RMY4, RMY5, RMY6, RMY7, RMY8, RMY9, RMY10, RMY11, RMY12, RMY13, RMY14, RMY15, RMY16, KRAY21, PMY4, KRAY37, PMY7*, and *KRAY40* strains were grown in SC-Leu⁻Ura⁻ medium overnight at 30 °C. Next day, the secondary culture was allowed to grow till mid-log phase and subsequently spotted on SC-Leu⁻Ura⁻ and SC-Leu⁻Ura⁻His⁻ plates. The plates were incubated at 30 °C for 4 days. Strains *NKY48, NKY50, RMY1, RMY9, KRAY21, PMY4*, and *PMY7* were used as a negative control.

### Coimmunoprecipitation

CoIP was performed using the previously used protocol (13). Briefly, *KRAY20, KRAY3, KRAY4, KRAY5, KRAY6*, *NFY24, KRAY54, KRAY55, KRAY56*, and *KRAY57* strains were grown in YPD medium at *A*_600_ till 0.5 at 30 °C. Cells were lysed and anti-Aha1 antibody was used to pull down Aha1, the IP was further checked for the presence of Hsp82. Strains *KRAY32, KRAY33, KRAY34, KRAY35, KRAY36*, *KRAY41*, *KRAY43, KRAY44*, *KRAY45*, *KRAY51*, and *KRAY53* along with *rad51Δ* strain (40) were grown till 0.5 *A*_600_ at 30 °C. Cells were lysed, Rad51 was immunoprecipitated by anti-Rad51 antibody and the presence Hsp82 and Aha1 were checked in the IP fraction.

### Western blotting

Western blotting was done to examine the protein levels in the coimmunoprecipitation and whole cell lysate samples as described previously (13). The primary antibodies used were mouse anti-Hsp90 antibody (Calbiochem), mouse anti-Nsp1 antibody (Abcam), rabbit anti-Aha1 antibody (Invitrogen), and rabbit anti-Rad51 antibody (GeneTex), at 1:5000 dilutions, the mouse anti-FLAG antibody (Abcam) was used at 1:2500 dilution. For secondary antibodies, horseradish peroxide-conjugated anti-rabbit antibody (Promega) and anti-mouse antibody (Promega) were used at 1:10,000 dilutions. The chemiluminescent detection system (Thermo Fisher Scientific) was used for developing the Western blots. The band intensities of multiple Western blots were quantified by using Image J software, and mean relative densities were plotted using GraphPad Prism 8.

### Hsp82 ATPase measurements

The Hsp82 ATPase activity was determined using a regenerative ATPase with NADH depletion (41, 42). A buffer containing phosphoenolpyruvate (5.33 mM), NADH (0.53 mM), pyruvate kinase (5.33U/ml), and lactate dehydrogenase (26.7 U/ml) in 40 mM Hepes, pH 7.5, 150 mM KCl, 5 mM MgCl2, and 6 mM β-ME was used. Hsp82 or mutant final monomer concentrations of 3 μM and 10 μM Aha1 were used for the experiment. The reaction was initiated by the addition of 2.5 mM ATP and the absorption at 340 nm was continuously recorded at 30 °C. After 30 min, radicicol was added at a concentration of 500 μM to inhibit the Hsp90 ATPase. As a control, cochaperones were tested in the absence of Hsp90 and the background activity was subtracted.

## Data availability

All other original data referred to in this article is held by the corresponding author´s institution and will be shared upon reasonable request; e-mail: sbtsl@uohyd.ac.in.

## Supporting information

This article contains supporting information (10, 13, 24, 27, 37, 40).

## Conflict of interest

The authors declare that they have no conflicts of interest with the contents of this article.
