# Supplementary material for: Identification of a chaperone-code responsible for Rad51-mediated genome repair
Source: J Biol Chem. 2024 May 3;300(6):107342. doi: 10.1016/j.jbc.2024.107342 (PMC11154708; doi:10.1016/j.jbc.2024.107342)
Supplement: Supplemental Figure S1 [file mmc1.pdf]

A

H. sapiens Hsp90α	18-	ETFAFQAEIAQLMSLIINTFYSNKEIFLRELIS	-50
G. gallus Hsp90β	13-	ETFAFQAEIAQLMSLIINTFYSNKEIFLRELIS	-45
S. cerevisiae Hsp82	4-	ETFEFQAEITQLMSLIINTVYSNKEIFLRELIS	-36
S. cerevisiae Hsc82	4-	ETFEFQAEITQLMSLIINTVYSNKEIFLRELIS	-36
G. gallus Hsp90α	17-	ETFAFQAEIAQLMSLIINTFYSNKEIFLRELIS	-49
H. sapiens Hsp90β	13-	ETFAFQAEIAQLMSLIINTFYSNKEIFLRELIS	-45
M. musculus Hsp90β	13-	ETFAFQAEIAQLMSLIINTFYSNKEIFLRELIS	-45
M. musculus Hsp90α	18-	ETFAFQAEIAQLMSLIINTFYSNKEIFLRELIS	-50

B

H. sapiens Hsp90α	292-	KTKPIWTRNPDDITNEEYGEFYKSLTNDWEDHL	-324
G. gallus Hsp90β	288-	KTKPIWTRNPDDITQEEYGEFYKSLTNDWEDHL	-321
S. cerevisiae Hsp82	272-	KTKPLWTRNPSDITQEEYNAFYKSISNDWEDPL	-304
S. cerevisiae Hsc82	268-	KTKPLWTRNPSDITQEEYNAFYKSISNDWEDPL	-300
G. gallus Hsp90α	288-	KTKPIWTRNPDDITNEEYGEFYKSLTNDWEDHL	-320
H. sapiens Hsp90β	284-	KTKPIWTRNPDDITQEEYGEFYKSLTNDWEDHL	-316
M. musculus Hsp90β	284-	KTKPIWTRNPDDITQEEYGEFYKSLTNDWEDHL	-316
M. musculus Hsp90α	293-	KTKPIWTRNPDDITNEEYGEFYKSLTNDWEEHL	-325

C

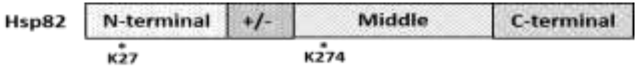

Supporting information Figure S1: (A) & (B) Multiple sequence alignment of Hsp90 isoforms (alpha and beta) from *Homo sapiens*, *Gallus gallus Saccharomyces cerevisiae* and *Mus musculus* showed that both the K27 and K274 residues (shown in bold and underlined) are conserved across the eukaryotes. (C) Schematic representation of Hsp82 domain structure illustrates the position of K27 in the N-terminal domain and K274 in the middle domain; (+/-) denotes the charged-linker region.
