# Supplementary material for: Identification of a chaperone-code responsible for Rad51-mediated genome repair
Source: J Biol Chem. 2024 May 3;300(6):107342. doi: 10.1016/j.jbc.2024.107342 (PMC11154708; doi:10.1016/j.jbc.2024.107342)
Supplement: Supplemental Table S1 [file mmc2.docx]

**Primers used in this study**

| **Primer**  **Name** | **Sequence** | **Purpose** |
| --- | --- | --- |
| OSB504 | 5’ ACA CCG TCT ATT CTA ACC AAG AAA TTT TCT TGA GAG 3’ | Forward primer used to generate *K27Qhsp82* mutation |
| OSB505 | 5’ ATC AGT TCT CTC AAG AAA ATT TCT TGG TTA GAA TAG 3’ | Reverse primer used to generate *K27Qhsp82* mutation |
| OSB506 | 5’ ACA CCG TCT ATT CTA ACA GAG AAA TTT TCT TGA GAG 3’ | Forward primer used to generate *K27Rhsp82* mutation |
| OSB507 | 5’ ATC AGT TCT CTC AAG AAA ATT TCT CTG TTA GAA TAG 3’ | Reverse primer used to generate *K27Rhsp82* mutation |
| OSB508 | 5’ AAG AAC TAA ACA AGA CTC AAC CTT TGT GGA CTA G 3’ | Forward primer used to generate *K274Qhsp82* mutation |
| OSB509 | 5’ TGG GTT TCT AGT CCA CAA AGG TTG AGT CTT GTT TAG 3’ | Reverse primer used to generate *K274Qhsp82* mutation |
| OSB510 | 5’ AAG AAC TAA ACA AGA CTA GAC CTT TGT GGA CTA G 3’ | Forward primer used to generate *K274Rhsp82* mutation |
| OSB511 | 5’ TGG GTT TCT AGT CCA CAA AGG TCT AGT CTT GTT TAG 3’ | Reverse primer used to generate *K274Rhsp82* mutation |
| OSB444 | 5’ GACGGATCCATGTCCGATAAAGTTATTAACCC 3’ | Forward primer used to amplify full length *SBA1* |
| OSB445 | 5’ GACGTCGACTTAAGCTTTCACTTCCGGCTC 3’ | Reverse primer used to amplify full length *SBA1* |
| OSB612 | 5’ GACGGATCCATGGCCATTGATTACTCTAAGTG 3’ | Forward primer used to amplify full length *CDC37* |
| OSB624 | 5’ GACGTCGACCTAGTCAACAGTGTCGGCAG 3’ | Reverse primer used to amplify full length *CDC37* |
| OSB561 | 5’ ATCTTGAGGTTATTCTTGAGAGATGACCAATTGGAG 3’ | Forward primer used to generate *K178Rhsp82* mutation |
| OSB562 | 5’ CCAAGTACTCCAATTGGTCATCTCTCAAGAATAACC 3’ | Reverse primer used to generate *K178Rhsp82* mutation |
| OSB376 | 5’ AGG GAA AGT TGA GCA CTG TAA TAC GCC GAA CAG ATT AAG CCG GAT CCC CGG GTT AAT TAA 3’ | Forward primer used to amplify *HDA1* knockout cassette |
| OSB377 | 5’ GAA GGT TGC CGA AAA AAA ATT ATT AAT GGC CAG TTT TTC CGA ATT CGA GCT CGT TTA AAC 3’ | Reverse primer used to amplify *HDA1* knockout cassette |
| OSB378 | 5’ TCC TCA CTT GCC ATT TTC CCG 3’ | Forward primer used to confirm *HDA1* knockout |
| OSB373 | 5’ CAT ACA AAA CAT TCG TGG CTA CAA CTC GAT ATC CGT GCA GCG GAT CCC CGG GTT AAT TAA 3’ | Forward primer used to amplify *RPD3* knockout cassette |
| OSB374 | 5’ TCA CAT TAT TTA TAT TCG TAT ATA CTT CCA ACT CTT TTT TGA ATT CGA GCT CGT TTA AAC 3’ | Reverse primer used to amplify *RPD3* knockout cassette |
| OSB375 | 5’ TGT GTC GCG GGC TGA ACT G 3’ | Forward primer used to confirm *RPD3* knockout |
| OSB275 | 5’ GTGTTACCCAGTTCACAATGG 3’ | Forward primer to amplify *KANMX* cassette from *Δaha1* strain |
| OSB720 | 5’ GACGCCATGGTCATCCTTGC 3’ | Reverse primer to amplify *KANMX* cassette from *Δaha1* strain |

**Yeast strains used in this study**

| **Strain** | **Genotype** | **Source** |
| --- | --- | --- |
| *P82a* | *MATa can1-100 ade2-1 his3-11,15 leu2-3,112 trp1 ura3-1 hsp82::LEU2 hsc82::LEU2 CEN pTGPD/P82* | (24) |
| *W303a* | *MATa 15 ade2-1 ura3-1,112 his3-11 trp1 leu2-3* | This Study |
| *KRAY20* | *MATa can1-100 ade2-1 his3-11,15 leu2-3,112 trp1 ura3-1 hsp82::LEU2 hsc82::LEU2, HSP82-CEN-HIS [pRS313/HSP82]* | This Study |
| *KRAY3* | *MATa can1-100 ade2-1 his3-11,15 leu2-3,112 trp1 ura3-1 hsp82::LEU2 hsc82::LEU2, K27Qhsp82-CEN-HIS [pRS313/K27Qhsp82]* | This Study |
| *KRAY4* | *MATa can1-100 ade2-1 his3-11,15 leu2-3,112 trp1 ura3-1 hsp82::LEU2 hsc82::LEU2, K27Rhsp82-CEN-HIS [pRS313/K27Rhsp82]* | This Study |
| *KRAY5* | *MATa can1-100 ade2-1 his3-11,15 leu2-3,112 trp1 ura3-1 hsp82::LEU2 hsc82::LEU2, K274Qhsp82-CEN-HIS [pRS313/K274Qhsp82]* | This Study |
| *KRAY6* | *MATa can1-100 ade2-1 his3-11,15 leu2-3,112 trp1 ura3-1 hsp82::LEU2 hsc82::LEU2, K27Rhsp82-CEN-HIS [pRS313/K274Rhsp82]* | This Study |
| *KRAY7* | *MATa can1-100 ade2-1 his3-11,15 leu2-3,112 trp1 ura3-1 hsp82::LEU2 hsc82::LEU2, K27QK274Qhsp82-CEN-HIS [pRS313/K27QK274Qhsp82]* | This Study |
| *KRAY8* | *MATa can1-100 ade2-1 his3-11,15 leu2-3,112 trp1 ura3-1 hsp82::LEU2 hsc82::LEU2, K27RK274Rhsp82-CEN-HIS [pRS313/K27RK274Rhsp82]* | This Study |
| *T101I* mutant | *MATa can1-100 ade2-1 his3-11,15 leu2-3,112 trp1 ura3-1 hsp82::LEU2 hsc82::LEU2 CEN pTGPD/T3-138* | (24) |
| *PJ69-4A* | *MATa trpl-901 leu2-3,112 ura3-52 his3-200 ga14∆ ga180∆ LYS2:: GAL1-HIS3 GAL2-ADE2 met2::GAL7-lacZ* | (37) |
| *NKY48* | *MATa trpl-901 leu2-3,112 ura3-52 his3-200 ga14∆ ga180∆ LYS2:: GAL1-HIS3 GAL2-ADE2 met2::GAL7-lacZ pGBDUC1 pGADC1* | (10) |
| *NKY50* | *MATa trpl-901 leu2-3,112 ura3-52 his3-200 ga14∆ ga180∆ LYS2:: GAL1-HIS3 GAL2-ADE2 met2::GAL7-lacZ pGBDUC1/HSP82 pGADC1* | (10) |
| *RMY1* | *MATa trpl-901 leu2-3,112 ura3-52 his3-200 ga14∆ ga180∆ LYS2:: GAL1-HIS3 GAL2-ADE2 met2::GAL7-lacZ pGBDUC1 pGADC1/SBA1* | This Study |
| *RMY2* | *MATa trpl-901 leu2-3,112 ura3-52 his3-200 ga14∆ ga180∆ LYS2:: GAL1-HIS3 GAL2-ADE2 met2::GAL7-lacZ pGBDUC1/HSP82 pGADC1/SBA1* | This Study |
| *RMY3* | *MATa trpl-901 leu2-3,112 ura3-52 his3-200 ga14∆ ga180∆ LYS2:: GAL1-HIS3 GAL2-ADE2 met2::GAL7-lacZ pGBDUC1/K27Qhsp82 pGADC1/SBA1* | This Study |
| *RMY4* | *MATa trpl-901 leu2-3,112 ura3-52 his3-200 ga14∆ ga180∆ LYS2:: GAL1-HIS3 GAL2-ADE2 met2::GAL7-lacZ pGBDUC1/K27Rhsp82 pGADC1/SBA1* | This Study |
| *RMY5* | *MATa trpl-901 leu2-3,112 ura3-52 his3-200 ga14∆ ga180∆ LYS2:: GAL1-HIS3 GAL2-ADE2 met2::GAL7-lacZ pGBDUC1/K274Qhsp82 pGADC1/SBA1* | This Study |
| *RMY6* | *MATa trpl-901 leu2-3,112 ura3-52 his3-200 ga14∆ ga180∆ LYS2:: GAL1-HIS3 GAL2-ADE2 met2::GAL7-lacZ pGBDUC1/K274Rhsp82 pGADC1/SBA1* | This Study |
| *RMY7* | *MATa trpl-901 leu2-3,112 ura3-52 his3-200 ga14∆ ga180∆ LYS2:: GAL1-HIS3 GAL2-ADE2 met2::GAL7-lacZ pGBDUC1/K27QK274Qhsp82 pGADC1/SBA1* | This Study |
| *RMY8* | *MATa trpl-901 leu2-3,112 ura3-52 his3-200 ga14∆ ga180∆ LYS2:: GAL1-HIS3 GAL2-ADE2 met2::GAL7-lacZ pGBDUC1/K27RK274Rhsp82 pGADC1/SBA1* | This Study |
| *RMY9* | *MATa trpl-901 leu2-3,112 ura3-52 his3-200 ga14∆ ga180∆ LYS2:: GAL1-HIS3 GAL2-ADE2 met2::GAL7-lacZ pGBDUC1 pGADC1/CDC37* | This Study |
| *RMY10* | *MATa trpl-901 leu2-3,112 ura3-52 his3-200 ga14∆ ga180∆ LYS2:: GAL1-HIS3 GAL2-ADE2 met2::GAL7-lacZ pGBDUC1/HSP82 pGADC1/CDC37* | This Study |
| *RMY11* | *MATa trpl-901 leu2-3,112 ura3-52 his3-200 ga14∆ ga180∆ LYS2:: GAL1-HIS3 GAL2-ADE2 met2::GAL7-lacZ pGBDUC1/K27Qhsp82 pGADC1/CDC37* | This Study |
| *RMY12* | *MATa trpl-901 leu2-3,112 ura3-52 his3-200 ga14∆ ga180∆ LYS2:: GAL1-HIS3 GAL2-ADE2 met2::GAL7-lacZ pGBDUC1/K27Rhsp82 pGADC1/CDC37* | This Study |
| *RMY13* | *MATa trpl-901 leu2-3,112 ura3-52 his3-200 ga14∆ ga180∆ LYS2:: GAL1-HIS3 GAL2-ADE2 met2::GAL7-lacZ pGBDUC1/K274Qhsp82 pGADC1/CDC37* | This Study |
| *RMY14* | *MATa trpl-901 leu2-3,112 ura3-52 his3-200 ga14∆ ga180∆ LYS2:: GAL1-HIS3 GAL2-ADE2 met2::GAL7-lacZ pGBDUC1/K274Rhsp82 pGADC1/CDC37* | This Study |
| *RMY15* | *MATa trpl-901 leu2-3,112 ura3-52 his3-200 ga14∆ ga180∆ LYS2:: GAL1-HIS3 GAL2-ADE2 met2::GAL7-lacZ pGBDUC1/K27QK274Qhsp82 pGADC1/CDC37* | This Study |
| *RMY16* | *MATa trpl-901 leu2-3,112 ura3-52 his3-200 ga14∆ ga180∆ LYS2:: GAL1-HIS3 GAL2-ADE2 met2::GAL7-lacZ pGBDUC1/K27RK274Rhsp82 pGADC1/CDC37* | This Study |
| *NFY24* | *MATa 15ade2-1 ura3-1,112 his3-11 trp1 leu2-3 AHA1::HIS* | (10) |
| *LS402* | *MATa leu2-3,112 trp1-1 can1-100 ura3-1 ade2-1 his3-11,15 [phi+] RAD51:: LEU2* | (40) |
| *KRAY32* | *MATa can1-100 ade2-1 his3-11,15 leu2-3,112 trp1 ura3-1 hsp82::LEU2 hsc82::LEU2, K27Qhsp82-CEN-HIS [pRS313/HSP82] RAD51-2µ-TRP [pTA/RAD51]* | This Study |
| *KRAY33* | *MATa can1-100 ade2-1 his3-11,15 leu2-3,112 trp1 ura3-1 hsp82::LEU2 hsc82::LEU2, K27Qhsp82-CEN-HIS [pRS313/K27Qhsp82] RAD51-2µ-TRP [pTA/RAD51]* | This Study |
| *KRAY34* | *MATa can1-100 ade2-1 his3-11,15 leu2-3,112 trp1 ura3-1 hsp82::LEU2 hsc82::LEU2, K27Rhsp82-CEN-HIS [pRS313/K27Rhsp82] RAD51-2µ-TRP [pTA/RAD51]* | This Study |
| *KRAY35* | *MATa can1-100 ade2-1 his3-11,15 leu2-3,112 trp1 ura3-1 hsp82::LEU2 hsc82::LEU2, K274Qhsp82-CEN-HIS [pRS313/K274Qhsp82] RAD51-2µ-TRP [pTA/RAD51]* | This Study |
| *KRAY36* | *MATa can1-100 ade2-1 his3-11,15 leu2-3,112 trp1 ura3-1 hsp82::LEU2 hsc82::LEU2, K274Rhsp82-CEN-HIS [pRS313/K274Rhsp82] RAD51-2µ-TRP [pTA/RAD51]* | This Study |
| *KRAY21* | *MATa trpl-901 leu2-3,112 ura3-52 his3-200 ga14∆ ga180∆ LYS2:: GAL1-HIS3 GAL2-ADE2 met2::GAL7-lacZ pGBDUC1 pGADC1/AHA1* | This Study |
| *PMY4* | *MATa trpl-901 leu2-3,112 ura3-52 his3-200 ga14∆ ga180∆ LYS2:: GAL1-HIS3 GAL2-ADE2 met2::GAL7-lacZ pGBDUC1/RAD51 pGADC1* | (13) |
| *KRAY37* | *MATa trpl-901 leu2-3,112 ura3-52 his3-200 ga14∆ ga180∆ LYS2:: GAL1-HIS3 GAL2-ADE2 met2::GAL7-lacZ pGBDUC1/RAD51 pGADC1/AHA1* | This Study |
| *PMY7* | *MATa trpl-901 leu2-3,112 ura3-52 his3-200 ga14∆ ga180∆ LYS2:: GAL1-HIS3 GAL2-ADE2 met2::GAL7-lacZ pGBDUC1 pGADC1/ RAD51* | (13) |
| *KRAY40* | *MATa trpl-901 leu2-3,112 ura3-52 his3-200 ga14∆ ga180∆ LYS2:: GAL1-HIS3 GAL2-ADE2 met2::GAL7-lacZ pGBDUC1/HSP82 pGADC1/ RAD51* | This Study |
| *KRAY41* | *MATa can1-100 ade2-1 his3-11,15 leu2-3,112 trp1 ura3-1 hsp82::LEU2 hsc82::LEU2, K178Rhsp82-CEN-HIS [pRS313/K178Rhsp82]* | This Study |
| *KRAY42* | *MATa 15ade2-1 ura3-1,112 his3-11 trp1 leu2-3 AHA1::HIS, RAD51-2µ-TRP [pTA/RAD51]* | This Study |
| *KRAY43* | *MATa 15ade2-1 ura3-1,112 his3-11 trp1 leu2-3 AHA1::HIS, RAD51-2µ-TRP [pTA/RAD51] HSP82-2µ-LEU [pLA/HSP82]* | This Study |
| *KRAY44* | *MATa can1-100 ade2-1 his3-11,15 leu2-3,112 trp1 ura3-1 hsp82::LEU2 hsc82::LEU2, K178Rhsp82-CEN-HIS [pRS313/K178Rhsp82] RAD51-2µ-TRP [pTA/RAD51]* | This Study |
| *KRAY45* | *MATa 15 ade2-1 ura3-1,112 his3-11 trp1 leu2-3, RAD51-2µ-TRP [pTA/RAD51] HSP82-2µ-LEU [pLA/HSP82]* | This Study |
| *NAY1* | *MATa can1-100 ade2-1 his3-11,15 leu2-3,112 trp1 ura3-1 hsp82::LEU2 hsc82::LEU2, HSP82-CEN-HIS [pRS313/HSP82] HDA1::TRP* | This Study |
| *KRAY48* | *MATa can1-100 ade2-1 his3-11,15 leu2-3,112 trp1 ura3-1 hsp82::LEU2 hsc82::LEU2, K27Qhsp82-CEN-HIS [pRS313/K27Qhsp82] HDA1::TRP* | This Study |
| *KRAY49* | *MATa can1-100 ade2-1 his3-11,15 leu2-3,112 trp1 ura3-1 hsp82::LEU2 hsc82::LEU2, K27Rhsp82-CEN-HIS [pRS313/K27Rhsp82] HDA1::TRP* | This Study |
| *NFY10* | *MATa 15ade2-1 ura3-1,112 his3-11 trp1 leu2-3 HDA1::TRP* | This Study |
| *KRAY50* | *MATa 15ade2-1 ura3-1,112 his3-11 trp1 leu2-3 RAD51-CEN-HIS [pRS313/RAD51]* | This Study |
| *KRAY51* | *MATa 15ade2-1 ura3-1,112 his3-11 trp1 leu2-3 RAD51-CEN-HIS [pRS313/RAD51] HSP82-2µ-LEU [pLA/HSP82]* | This Study |
| *KRAY52* | *MATa 15ade2-1 ura3-1,112 his3-11 trp1 leu2-3 HDA1::TRP RAD51-CEN-HIS [pRS313/RAD51]* | This Study |
| *KRAY53* | *MATa 15ade2-1 ura3-1,112 his3-11 trp1 leu2-3 HDA1::TRP RAD51-CEN-HIS [pRS313/RAD51] HSP82-2µ-LEU [pLA/HSP82]* | This Study |
| *KRAY54* | *MATa 15ade2-1 ura3-1,112 his3-11 trp1 leu2-3 HSP82-2µ-LEU [pLA/HSP82]* | This Study |
| *KRAY55* | *MATa 15ade2-1 ura3-1,112 his3-11 trp1 leu2-3 HDA1::TRP HSP82-2µ-LEU [pLA/HSP82]* | This Study |
| *NFY11* | *MATa 15ade2-1 ura3-1,112 his3-11 trp1 leu2-3 RPD3::HIS* | This Study |
| *KRAY56* | *MATa 15ade2-1 ura3-1,112 his3-11 trp1 leu2-3 RPD3::HIS HSP82-2µ-LEU [pLA/HSP82]* | This Study |
| *NFY12* | *MATa 15ade2-1 ura3-1,112 his3-11 trp1 leu2-3 HDA1::TRP RPD3::HIS* | This Study |
| *KRAY57* | *MATa 15ade2-1 ura3-1,112 his3-11 trp1 leu2-3 HDA1::TRP RPD3::HIS HSP82-2µ-LEU [pLA/HSP82]* | This Study |
| *NA3* | *MATa-inc ade2 ade3::GALHO ura3HOcs ---TRP1 ---URA3 leu2-3,112 his3-11,13 trp1-1 lys2::ura3::HOcs-inc* | (27) |
| *NA3rad51∆* | *MATa-inc ade2 ade3::GALHO ura3HOcs ---TRP1 ---URA3 leu2-3,112 his3-11,13 trp1-1 lys2::ura3::HOcs-inc rad51::kanMX* | (27) |
| *AKG1* | *MATa-inc ade2 ade3::GALHO ura3HOcs ---TRP1 ---URA3 leu2-3,112 his3-11,13 trp1-1 lys2::ura3::HOcs-inc aha1::KanMX* | This study |
